# WNT4 mediates estrogen receptor signaling and endocrine resistance in invasive lobular carcinoma cell lines

**DOI:** 10.1186/s13058-016-0748-7

**Published:** 2016-09-20

**Authors:** Matthew J. Sikora, Britta M. Jacobsen, Kevin Levine, Jian Chen, Nancy E. Davidson, Adrian V. Lee, Caroline M. Alexander, Steffi Oesterreich

**Affiliations:** 1 Women’s Cancer Research Center, University of Pittsburgh, Pittsburgh, PA USA; 2 Department of Pharmacology and Chemical Biology, University of Pittsburgh, Pittsburgh, PA USA; 3 Present address: Department of Pathology, University of Colorado – Anschutz Medical Campus, Mail Stop 8104, Research Complex 1 South, Room 5117, 12801 East 17th Avenue, Aurora, CO 80045 USA; 4 Department of Pathology, University of Colorado – Anschutz Medical Campus, Aurora, CO USA; 5 Department of Pathology, University of Pittsburgh, Pittsburgh, PA USA; 6 McArdle Laboratory for Cancer Research, University of Wisconsin-Madison, Madison, WI USA

**Keywords:** Breast cancer, Lobular carcinoma, Estrogen receptor, Endocrine therapy, Estradiol, Wnt signaling, Endocrine resistance

## Abstract

**Background:**

Invasive lobular carcinoma (ILC) of the breast typically presents with clinical biomarkers consistent with a favorable response to endocrine therapies, and over 90 % of ILC cases express the estrogen receptor (ER). However, a subset of ILC cases may be resistant to endocrine therapies, suggesting that ER biology is unique in ILC. Using ILC cell lines, we previously demonstrated that ER regulates a distinct gene expression program in ILC cells, and we hypothesized that these ER-driven pathways modulate the endocrine response in ILC. One potential novel pathway is via the Wnt ligand WNT4, a critical signaling molecule in mammary gland development regulated by the progesterone receptor.

**Methods:**

The ILC cell lines MDA-MB-134-VI, SUM44PE, and BCK4 were used to assess *WNT4* gene expression and regulation, as well as the role of WNT4 in estrogen-regulated proliferation. To assess these mechanisms in the context of endocrine resistance, we developed novel ILC endocrine-resistant long-term estrogen-deprived (ILC-LTED) models. ILC and ILC-LTED cell lines were used to identify upstream regulators and downstream signaling effectors of WNT4 signaling.

**Results:**

ILC cells co-opted WNT4 signaling by placing it under direct ER control. We observed that ER regulation of *WNT4* correlated with use of an ER binding site at the *WNT4* locus, specifically in ILC cells. Further, WNT4 was required for endocrine response in ILC cells, as *WNT4* knockdown blocked estrogen-induced proliferation. ILC-LTED cells remained dependent on WNT4 for proliferation, by either maintaining ER function and *WNT4 *regulation or uncoupling *WNT4* from ER and upregulating *WNT4* expression. In the latter case, *WNT4* expression was driven by activated nuclear factor kappa-B signaling in ILC-LTED cells. In ILC and ILC-LTED cells, WNT4 led to suppression of *CDKN1A*/p21, which is critical for ILC cell proliferation. *CDKN1A* knockdown partially reversed the effects of *WNT4* knockdown.

**Conclusions:**

WNT4 drives a novel signaling pathway in ILC cells, with a critical role in estrogen-induced growth that may also mediate endocrine resistance. WNT4 signaling may represent a novel target to modulate endocrine response specifically for patients with ILC.

**Electronic supplementary material:**

The online version of this article (doi:10.1186/s13058-016-0748-7) contains supplementary material, which is available to authorized users.

## Background

Invasive lobular carcinoma (ILC) represents the second most common subtype of breast cancer, and overall it is the sixth most common cancer diagnosis in women in the United States [1]. ILC cases typically present with favorable biomarkers, as >90 % of ILCs are estrogen receptor (ER)-positive and progesterone receptor (PR)-positive, <10 % are human epidermal growth factor receptor 2 (HER2)-positive, and the majority are low Ki-67-positive [1–5]. On the basis of these biomarkers, ILCs are an archetype of the luminal A molecular subtype, and patients with ILC may be expected to have favorable outcomes when treated with adjuvant endocrine therapy. However, recent retrospective analyses of the BIG 1-98 trial [6] and ABCSG-8 trial [7] suggest that, compared with patients with similar invasive ductal carcinomas (IDCs), a subset of patients with ILC may in fact have poorer outcomes with endocrine therapy. Improved understanding of ER signaling, endocrine response, and the development of endocrine resistance in ILC is critical to improving patient outcomes.

We previously reported a study of unique ER-mediated gene expression and signaling in ILC model systems using gene expression microarrays coupled with ER chromatin immunoprecipitation sequencing (ChIP-seq) [8]. In this study, the most strongly induced ILC-specific ER target gene was the Wnt ligand *WNT4*. Additionally, ChIP-seq identified an ILC-specific estrogen receptor binding site (ERBS) at the *WNT4* locus, approximately 1.5 kb downstream from the *WNT4* transcription start site, an evolutionarily conserved region [9] that contains two predicted estrogen response elements (EREs) (diagrammed in Additional file 1: Figure S1). These observations suggest that direct ER binding at this site may be responsible for estrogen-induced *WNT4* expression. Importantly, ILC cells may be co-opting *WNT4* regulation by placing it under ER control, as Wnt4 is a transcriptional target and downstream effector of PR signaling in the murine adult mammary gland [10–14]. In this context, Wnt4 is critical to maintaining a mammary progenitor cell population (reviewed by Brisken et al. [15]). Decreased progenitor cell potential during parity (and subsequent parity-induced breast cancer protection) is linked to downregulation of *Wnt4* [11], but progenitor cell proliferation is rescued by *Wnt4* induction [16] or exogenous WNT4 [11]. On the basis of these observations, we hypothesized that WNT4 may play a critical role in estrogen-regulated phenotypes in ILC.

To test this hypothesis, we assessed regulation and expression of *WNT4*, WNT4 signaling, and WNT4-mediated phenotypes in ILC- and IDC-derived breast cancer cell lines. In addition, we established a series of long-term estrogen-deprived (LTED) endocrine-resistant variants of the ILC cell lines MDA-MB-134-VI (MM134) and SUM44PE (44PE), and examined the role of WNT4 in endocrine resistance in these models. Our findings suggest that WNT4 signaling is a putative target to modulate endocrine response and combat endocrine resistance for ILC.

## Methods

### Cell culture

MCF-7 and T47D (American Type Culture Collection [ATCC], Manassas, VA, USA) cells were maintained as described elsewhere [17]. MM134 (ATCC) and 44PE (Asterand Bioscience, Detroit, MI, USA) cells were maintained as described previously [8]. MDA-MB-330 cells (MM330; ATCC) were maintained as described for MM134. HCC1428 and HT1080 cells (ATCC) were maintained in DMEM (11965; Life Technologies, Carlsbad, CA, USA) + 10 % FBS (26140; Life Technologies). BCK4 (University of Colorado Anschutz) was maintained as described elsewhere [18]. All lines were incubated at 37 °C in 5 % CO_2_. Cell lines were authenticated annually by polymerase chain reaction (PCR)-restriction fragment length polymorphism analyses at the University of Pittsburgh Cell Culture and Cytogenetics Facility and confirmed to be mycoplasma-negative. Authenticated cells were in continuous culture for <6 months. Cells were hormone-deprived using charcoal-stripped FBS (CSS) (12676, lot 1176965; Life Technologies), as described previously [17], in phenol red-free improved minimum essential medium (IMEM) + 10 % CSS (2 % CSS for SUM44PE only). This single lot of CSS was used for all experiments and was confirmed to have complete hormone deprivation [19].

17β-Estradiol (E2) and 4-hydroxytamoxifen (4-OHT) were obtained from Sigma-Aldrich (St. Louis, MO, USA); other compounds were obtained from Tocris Biosciences (Bristol, UK). E2, 4-OHT, ICI 182,780 (ICI), and progesterone (P4) were dissolved in ethanol; RU486 (RU, mifepristone), BMS-345541 (BMS), staurosporine (STS), *endo*-IWR1 (IWR), and JW 67 (JW) were dissolved in dimethyl sulfoxide.

### Proliferation and viability assays

For cellular proliferation assays, we used the FluoReporter double-stranded DNA quantitation kit (F2692; Life Technologies) according to the manufacturer’s instructions. Cell death was assessed using CellTox Green (G8741; Promega, Madison, WI, USA) according to the manufacturer’s instructions. For each assay, cells were plated in 96-well plates and allowed to attach overnight prior to the indicated treatments. Fluorescence was assessed using a VICTOR X4 plate reader (PerkinElmer, Waltham, MA, USA). For assays, points and/or bars represent the mean of five or six biological replicates ± SD.

### RNA interference

Small interfering RNAs (siRNAs) were reverse-transfected using Lipofectamine RNAiMAX reagent (Life Technologies) according to the manufacturer’s instructions. A list of constructs used in this study is available in Additional file 2. Notably, the efficacy of *WNT4* knockdown varied across commercially available constructs. The extent of knockdown correlated with effects on growth (Additional file 3: Figure S2). The reagent indicated (Additional file 2) outperformed other reagents tested (additional details available on request).

### Gene expression analyses

For RNA extractions, we used the illustra RNAspin Mini Kit (GE Healthcare Life Sciences, Little Chalfont, UK) or the RNeasy Mini Kit (QIAGEN, Hilden, Germany). For complementary DNA conversion, we used iScript master mix (Bio-Rad Laboratories, Hercules, CA, USA), and for quantitative PCR (qPCR) reactions, we used SsoAdvanced SYBR Green Master Mix (Bio-Rad Laboratories) on a CFX384 thermocycler (Bio-Rad Laboratories), according to the manufacturer’s instructions. Expression data were normalized to *RPLP0*. Primer sequences are available in Additional file 2.

### Chromatin immunoprecipitation

Cells were hormone-deprived as described above prior to treatment with 0.1 % EtOH, 1 nM E2, 1 μM ICI, 100 nM P4, or 1 μM RU486 for 45 minutes. ChIP experiments were performed as described previously [20] with minor modifications:

1. Nuclei were extracted prior to sonication by resuspending the fixed cell pellet in nuclei preparation buffer (5 mM 1,4-piperazinediethanesulfonic acid (PIPES), 85 mM KCl [pH 8.0] + 0.5 % Nonidet P-40 + protease inhibitor) with rotation at 4 °C for 30 minutes. Nuclei were then pelleted and lysed and/or sonicated as described.
2. SDS was omitted from buffers Tris-sucrose-ethylenediaminetetraacetic acid I (TSEI) and TSEII.
3. Carrier molecules were added during immunoprecipitation [21].

In immunoprecipitation experiments, we used ERα (HC-20) and rabbit immunoglobulin G (sc2027) antibodies (Santa Cruz Biotechnologies, Dallas, TX, USA). PCR was performed as described above, normalized to percentage input. Primer sequences are available in Additional file 2.

### Long-term estrogen deprivation

Endocrine-resistant variants of MM134 and 44PE were generated by maintaining cells in hormone-deprived conditions using IMEM + 10 % CSS. As 44PE cells are only modestly hormone-responsive and their basal medium has minimal hormone content, we first subderived cells in fully hormone-replete conditions by maintaining SUM44PE as described for MM134 (DMEM/L-15 + 10 % FBS) for 3 months. The resulting variant, termed SUM44/F, has an increased proliferative response to E2 (Additional file 4: Figure S3). To generate ILC-LTED lines, MM134 and SUM44/F were hormone-deprived as described above and subsequently plated in a 6-well plate. Each well was maintained independently over 6–12 months until cells could be passaged routinely; this generated four LTED MM134 and two LTED SUM44/F lines.

### Immunoblotting

SDS-PAGE was performed using standard methods. Proteins were transferred to polyvinylidene difluoride (PVDF) membranes for Western blot analysis using chemiluminescence detection. Antibodies were used according to the manufacturers’ recommendations: ERα (clone 6 F11; Leica Biosystems, Buffalo Grove, IL, USA), p65 (8242; Cell Signaling Technology, Danvers, MA, USA), phospho-p65 (serine 536, CS 3033; Cell Signaling Technology), RelB (CS 4922; Cell Signaling Technology), c-Rel (CS 4727; Cell Signaling Technology), NFKB1 (p105/p50, CS 12540; Cell Signaling Technology), p21 (CS 2946; Cell Signaling Technology), DVL2 (CS 3216; Cell Signaling Technology), DVL3 (CS 3218; Cell Signaling Technology), and tubulin (T9026; Sigma-Aldrich).

### Transcription factor response element reporter assays

A targeted screen for transcription factor activity was performed using the Cignal 45-Pathway Reporter Array System (QIAGEN). Plasmids were reverse-transfected using Attractene (QIAGEN). The following day, cells were treated with 0.01 % EtOH or 100 nM ICI. All conditions were performed in biological triplicate. Cells were assayed for reporter activity 42 h posttreatment using the Dual-Luciferase Reporter Assay System (Promega).

For canonical Wnt signaling reporter assays, we used TOP and Renilla plasmids, a kind gift from the Monga laboratory (University of Pittsburgh). Wnt expression plasmids were obtained from the Open Source Wnt Project (Addgene, Cambridge, MA, USA). The plasmid kit was a gift from Marian Waterman, David Virshup, and Xi He (kit 1000000022; Addgene). Plasmids were cotransfected using Lipofectamine LTX and PLUS reagent (Life Technologies). Cells were assayed for reporter activity 24 h posttransfection using the Promega Dual-Luciferase Reporter Assay System.

### Statistical analyses

Curve-fitting and statistical analyses for in vitro studies were performed using Prism version 5.04 software (GraphPad Software, La Jolla, CA, USA). For in silico analyses, we used expression values derived from The Cancer Genome Atlas (TCGA) [22] breast cancer cases and normal tissue (in units of transcripts per million) downloaded from the Gene Expression Omnibus database [GEO:GSE62944] [23]. PAM50 subtypes for the TCGA tumors were defined using the “genefu” package in R (version 2.4.2). Briefly, 50:50 distributions of ER^+^/ER^−^ tumors were sampled for the median centering step, and subtypes were assigned to all tumors. This process was repeated 100 times, and the consensus subtype for each tumor was taken. Molecular Taxonomy of Breast Cancer International Consortium (METABRIC) [24] data were downloaded from the Synapse software platform (syn1688369; Sage Bionetworks, Seattle, WA, USA). Microarray probes were selected for individual genes based on the probe set with the highest interquartile range.

## Results

### WNT4 is necessary for ILC cell proliferation in culture

**Fig. 1:**
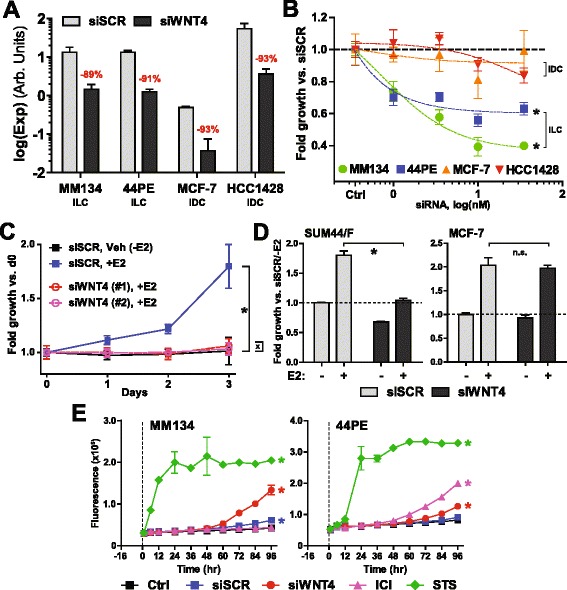
WNT4 is necessary for estrogen-induced growth in invasive lobular carcinoma (ILC) cells. **a** Breast cancer cell lines (BCCLs) were reverse-transfected with 10 nM siWNT4 or siSCR (Scrambled siRNA control) pools. *WNT4 *expression was assessed by quantitative polymerase chain reaction. Bars represent mean of biological triplicate ± SD. *p* < 0.05 for each siSCR vs siWNT4 (*t* test). **b** BCCLs were transfected as in (**a**) with increasing concentrations of small interfering RNA (siRNA), and proliferation was assessed 6 days posttransfection. siWNT4-treated cell proliferation was normalized to siSCR of equivalent concentration. *p < 0.01 by analysis of variance (ANOVA) of siRNA effect (siSCR vs siWNT4). **c** MDA-MB-134-VI (MM134) cells were hormone-deprived and reverse-transfected with siSCR or individual siWNT4 constructs. Cells were then treated with 100 pM 17β-estradiol (E2) or 0.01 % EtOH approximately 16 h posttransfection, and proliferation was assessed at the indicated time posttreatment. *,*p* < 0.0001 by ANOVA of E2 effect (siSCR without E2 vs with E2). x *p* = n.s. by ANOVA of E2 effect (siSCR without E2 vs either siWNT4). **d** Cells were treated as in (**c**), and proliferation was assessed 6 days posttreatment. *p < 0.05 for condition with E2 siSCR vs siWNT4 (*t* test). *n.s.* Not significant. **e** BCCLs were reverse-transfected with 10 nM siWNT4 or siSCR. The following day (after approximately 16 h), cells were treated with CellTox Green dye and 1 μM ICI 182,780 (fulvestrant; ICI) or staurosporine (STS) as indicated. Increased fluorescence represents accumulation of nonviable cells. Time points represent repeated measures of the same initial cell populations. *p < 0.05 by two-way ANOVA vs control, treatment effect. *IDC* Invasive ductal carcinoma, *44PE* SUM44PE cell line

To determine whether WNT4 is necessary for breast cancer cell proliferation, we used siRNA to knock down *WNT4* expression in breast cancer cell lines (BCCLs). *WNT4* knockdown was performed in the ILC cell lines MDA-MB-134-VI (MM134) and SUM44PE (44PE) and compared with IDC cell lines MCF-7 and HCC1428. Notably, MCF-7 cells expressed more than tenfold less *WNT4* than ILC lines, while HCC1428 was the only ER-positive BCCL with higher *WNT4* expression than MM134 [25, 26]; this was confirmed by qPCR (Fig. 1a). In all four BCCLs, siRNA targeting *WNT4* (siWNT4) produced about 90 % knockdown (Fig. 1a). siWNT4 suppressed the growth of both MM134 and 44PE cells (by approximately 60 % and 40 %, respectively) (Fig. 1b). However, growth suppression was not observed in MCF-7 or HCC1428 (Fig. 1b).

We further assessed whether WNT4 was specifically necessary for estrogen-induced growth. The growth of MM134 plus E2 was completely suppressed by either of two individual siWNT4 constructs. Growth was equivalent to the absence of estrogen (Fig. 1c). This was also observed in SUM44/F (a 44PE variant with increased endocrine response; see the Methods section above and Additional file 4: Figure S3), but siWNT4 had no effect on estrogen-induced growth in MCF-7 (Fig. 1d). Importantly, the effect of siWNT4 on cell growth is likely immediately due to an inhibition of proliferation, as cell death following siWNT4 was not observed until more than 3 days post-*WNT4* knockdown (Fig. 1e). These data suggest that WNT4 may be required for the proliferation of ILC cells but not IDC cells, and that estrogen-induced proliferation in ILC cells requires WNT4.

### Estrogen regulates WNT4 expression via ER binding at the WNT4 ERBS

**Fig. 2:**
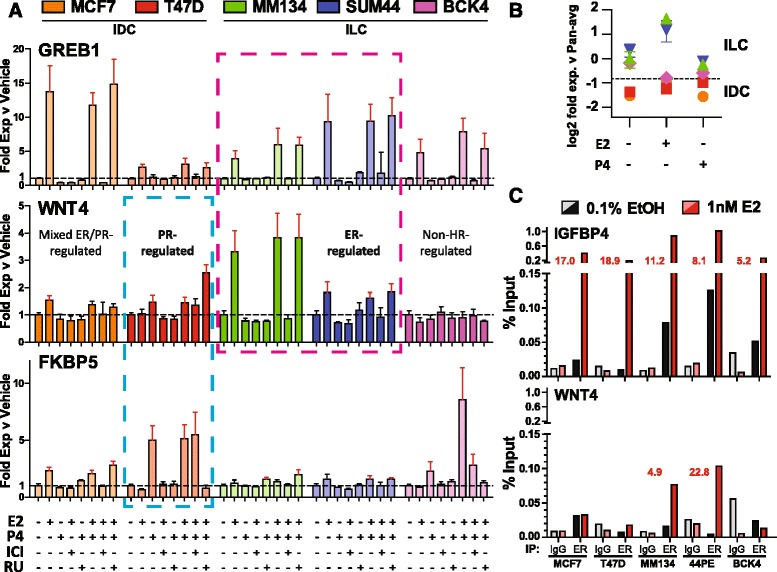
Estrogen regulation of *WNT4* correlates with estrogen receptor (ER) binding at the *WNT4* estrogen receptor binding site (ERBS). **a** Breast cancer cell lines (BCCLs) were hormone-deprived and treated in biological triplicate with vehicle (0.2 % EtOH), 1 nM 17β-estradiol (E2), 100 nM progesterone (P4), 1 μM ICI 182,780 (fulvestrant; ICI), or 1 μM RU486 (RU), as indicated. RNA was harvested 24 h posttreatment. Bars represent mean ± SD as fold change vs vehicle control; *red error bars* indicate analysis of variance (Dunnett’s multiple comparisons test) vs vehicle control (*p* < 0.05). **b** Data from (**a**) were normalized to a pan-average of all samples across BCCLs tested. **c** BCCLs were hormone-deprived and treated as indicated for 45 minutes. Chromatin immunoprecipitation (ChIP) was performed as described in the Methods section. *Red* values indicate fold enrichment for E2 vs vehicle in ER ChIP. The data were derived from a single experiment but are representative of two or three experiments. *SUM44* SUM44PE cell line, *HR* Hormone receptor, *IDC* Invasive ductal carcinoma, *IgG* Immunoglobulin G, *ILC* Invasive lobular carcinoma, *PR* Progesterone receptor

On the basis of potential roles for both ER and PR in regulating *WNT4* expression, we used a series of ER-positive ILC (MM134, 44PE, BCK4) and IDC (MCF-7, T47D) BCCLs to further investigate *WNT4* regulation. Expression of *WNT4* in response to combinations of E2, P4, and antihormones was compared with *GREB1* (ER target gene) and *FKBP5* (PR target gene). As shown in Fig. 2a, GREB1 expression was induced by E2 and reversed by ICI in all lines, whereas FKBP5 was only P4-induced in the strongly PR-positive lines T47D and BCK4. Other BCCLs tested were PR-weak or negative. These experiments confirmed that *WNT4* expression was solely ER-regulated in MM134 and 44PE; P4 and the PR antagonist RU486 had no effect, whereas *WNT4* expression was E2-induced and ICI-reversed. In BCK4, neither E2 nor P4 induced *WNT4*. We also observed ER regulation of *WNT4* in the ILC cell line MM330 (Additional file 5: Figure S4a), which we recently characterized as having functional ER signaling (M. J. Sikora et al., unpublished data). In contrast, IDC cells maintained complete or partial PR control of *WNT4* expression. *WNT4* was weakly E2-induced in MCF-7, but P4 and/or RU cotreatment reduced expression, suggesting that *WNT4* may be ER/PR-coregulated in MCF-7. In T47D, *WNT4* expression was solely PR-regulated, as P4 induced expression but E2 had no effect. Thus, IDC cells may maintain *WNT4* under complete or partial PR control, similarly to the normal adult mammary gland, whereas some ILCs switch *WNT4* from PR to ER regulation.

Interestingly, though we detected ER regulation of *WNT4* in MCF-7 in qPCR-based analysis, this was not observed in our prior microarray analyses [8]. In retrospect, *WNT4* expression values failed to pass minimum microarray signal thresholds in the public datasets used. Consistent with this, overall *WNT4* expression was very low in IDC vs ILC lines (Fig. 2b).

Using ChIP-qPCR, we then assessed whether *WNT4* regulation by the ER correlated with ER binding at the WNT4 ERBS (Additional file 1: Figure S1) (Fig. 2c). As a control, we assessed ER binding at a canonical ERBS at *IGFBP4*, and we found that E2 induced strong ER binding in all five BCCLs, consistent with activation of canonical ER-mediated transcription. However, E2 induced binding at *WNT4* only in ILC cells with ER-specific regulation of *WNT4*, namely MM134 and 44PE. This is consistent with the hypothesis that ER directly regulates *WNT4* expression via ER binding at the WNT4 ERBS in ILC cells.

Recently, Mohammed et al. reported a functional ER-PR interaction that modified genomic binding in breast cancer cell lines [27]. As ER and PR may interact to regulate *WNT4* in MCF-7 cells (Fig. 2a), we investigated the ChIP-seq data reported by Mohammed et al. These data identified weak, inconsistent ER binding at the WNT4 ERBS and a P4-induced ER binding site about 30 kb upstream from *WNT4* in MCF-7 cells (Additional file 5: Figure S4b). We assessed ER binding at these loci with E2 with or without P4, and we did not detect P4-induced changes in ER binding at *IGFBP4* or *WNT4*. Additionally, we did not detect binding at the upstream site (Additional file 5: Figure S4c). These data do not identify a direct link between P4-induced changes in ER binding and changes in *WNT4* regulation.

### Endocrine-resistant ILC cell lines upregulate WNT4 or maintain ER regulation of WNT4

We hypothesized that because WNT4 plays a critical role in estrogen-induced proliferation in ILC, it may play a similar role in endocrine resistance. To model endocrine resistance during aromatase inhibitor therapy in ILC, we generated LTED variants of MM134 (134:L/A, L/B, L/D, and L/E; collectively referred to as *134:LTED*) and 44PE (44:L/A and L/B; collectively referred to as *44:LTED*) (Additional file 6: Figure S5a). All LTED lines remained ER-positive, as determined by immunoblot analysis. Each of the 134:LTED lines had reduced ER vs parental cells, whereas both 44:LTED lines had increased ER vs 44PE and SUM44/F (Additional file 6: Figure S5b). Despite retaining ER expression, 134:LTED lines were no longer endocrine-responsive. Neither E2 nor 4-OHT induced proliferation, and ICI had no effect in any 134:LTED line (Additional file 6: Figure S5c). Conversely, though E2 did not induce proliferation in the 44:LTED lines, each was growth-inhibited by both 4-OHT and ICI, suggesting that ER activation is maintained in 44:LTED cells despite the absence of exogenous steroid hormones (Additional file 6: Figure S5c).

**Fig. 3:**
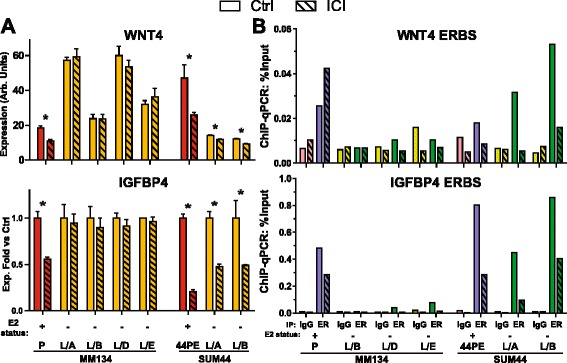
Expression and regulation of *WNT4* in invasive lobular carcinoma endocrine-resistant long-term estrogen-deprived (ILC-LTED) cells correlates with use of the *WNT4* estrogen receptor binding site (ERBS). **a** Breast cancer cell lines (BCCLs) in their respective standard conditions were treated in biological triplicate with 0.1 % EtOH or 1 μM ICI 182,780 (fulvestrant; ICI). RNA was harvested 24 h posttreatment. Bars represent mean ± SD. *p < 0.05 for vehicle control vs ICI (*t* test). **b** BCCLs were treated as in (**a**) for 60 minutes. Chromatin immunoprecipitation (ChIP) was performed as described in the Methods section. Data derived from single experiment, but are representative of duplicate experiments. “E2 status” denotes the hormone status of the experimental culture medium. ^+^FBS-containing medium; ^−^Charcoal-stripped FBS-containing medium. *qPCR* Quantitative polymerase chain reaction, *E2* 17β-Estradiol, *MM134* MDA-MB-134-VI, *SUM44* SUM44PE cell line

We next examined expression and ER regulation of *WNT4* in ILC-LTED cells. Each 134:LTED cell line upregulated *WNT4* vs parental MM134 cells in hormone-replete conditions (Fig. 3a, top), but ICI treatment did not affect expression in any 134:LTED line. Conversely, 44:LTED had decreased *WNT4* expression vs 44PE; however, expression was reduced by ICI in 44:LTED. This suggests that ER regulation of *WNT4* is maintained in 44:LTED despite hormone deprivation. Estrogen regulation of *WNT4* was paralleled by similar observations with *IGFBP4* (Fig. 3a, bottom) and *GREB1* (Additional file 7: Figure S6a). ICI had no effect on *IGFBP4* or *GREB1* expression in 134:LTED, but it reduced their expression in 44:LTED. These data parallel the proliferative endocrine responses described above (Additional file 6: Figure S5c). Taken together, *WNT4* upregulation independent of ER, or maintenance of ER control of *WNT4*, is a shared feature across this series of six endocrine-resistant ILC lines.

We then assessed ER binding at the WNT4 ERBS, based on the changes in *WNT4* expression and regulation in ILC-LTED cells. Shown in Fig. 3b (top), consistent with the loss of ER regulation of *WNT4*, we observed minimal or no ER binding at this locus in 134:LTED. However, strong ER binding was observed at the WNT4 ERBS in 44:LTED, paralleling maintained ER regulation. This binding could be ablated with ICI. Similar results were observed at the IGFBP4 ERBS (Fig. 3b, bottom). These results are consistent with the hypothesis that ER binding at the WNT4 ERBS is required for ER regulation of *WNT4*, as the 44:LTED lines maintained ER binding at this site, whereas the 134:LTED lines had uncoupled *WNT4* from the ER and no longer used the WNT4 ERBS.

### WNT4 is critical for proliferation in LTED models

**Fig. 4:**
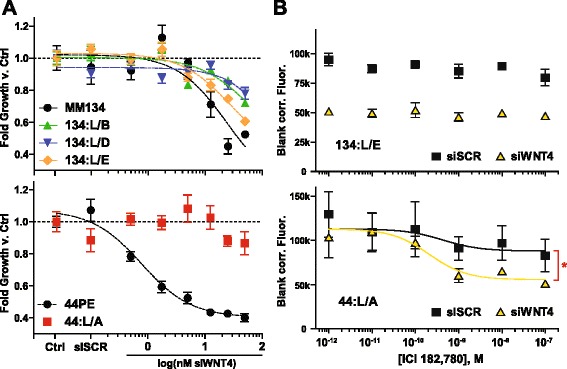
WNT4 dependence in invasive lobular carcinoma endocrine-resistant long-term estrogen-deprived cells (ILC-LTED) cells is linked to endocrine response context. **a** Breast cancer cell lines were reverse-transfected with increasing concentrations of siWNT4 or 12.5 nM siSCR, or were mock-transfected (Ctrl). Growth was assessed 6 days posttransfection. siSCR was toxic in 134:L/A and 44:L/B, and these lines were not included in analyses or future small interfering RNA (siRNA) experiments. **b** Cells were reverse transfected with 35 nM siRNA and treated with increasing concentrations of ICI 182,780 (fulvestrant; ICI) approximately 16 h posttransfection. Growth was assessed 6 days posttreatment. *p < 0.05; bottom of nonlinear regression for siSCR vs siWNT4. *44PE* SUM44PE cell line, *MM134* MDA-MB-134-VI

The maintenance of *WNT4* expression or regulation in the LTED phenotype suggests that ILC-LTED cells may remain dependent on WNT4 signaling. To test this, we used siRNA as described above to knock down *WNT4*. The 134:LTED lines remained growth-inhibited by siWNT4, but the siWNT4 was less effective in suppressing growth than in the parental MM134 cells (Fig. 4a, top). 134:L/E was most sensitive, with growth suppressed about 40 %. However, unlike 134:LTED, 44:LTED cells were completely resistant to siWNT4 relative to 44PE (Fig. 4a, bottom). This correlates with the increased *WNT4* expression in 134:LTED but decreased expression in 44:LTED (Fig. 3). Because 44:LTED displayed maintained ER activity and *WNT4* regulation, we hypothesized that siWNT4 combined with ICI may potentiate growth suppression. As shown in Fig. 4b (top), siWNT4 alone inhibited proliferation of 134:L/E, but ICI treatment had no effect, consistent with lack of endocrine response. Conversely, 44:L/A was resistant to siWNT4, but growth was modestly inhibited by ICI alone. Combining siWNT4 with ICI increased growth suppression beyond either treatment alone (Fig. 4b, bottom). We also examined combining siWNT4 with fibroblast growth factor receptor (FGFR) inhibition, as parental ILC cells are codependent on ER and FGFR1 [8]. ILC-LTED cells remained sensitive to the FGFR inhibitor PD173074, but the relative effect was equivalent with or without siWNT4 (Additional file 7: Figure S6b), suggesting that additional signaling pathways were activated during LTED to maintain viability. Thus, ILC-LTED cells require WNT4 to maintain proliferation, but the context of this dependence is based on the endocrine responsiveness of the cells targeted.

### Activated nuclear factor kappa-B in LTED models regulates WNT4-CDKN1A/p21 pathway

A defining feature of ILC is E-cadherin loss associated with dysfunction or loss of catenin proteins (e.g., β-catenin loss in ILC vs IDC [28]; Additional file 8: Figure S7a). Additionally, though WNT4 can activate β-catenin in some contexts, it is typically considered a noncanonical Wnt ligand [29, 30]. Coupled with the lack of β-catenin protein in ILC [31, 32], it is unlikely that WNT4 can activate canonical, β-catenin-dependent Wnt signaling in ILC cells. We confirmed this using the TOPFlash reporter in MM134 cells (Additional file 8: Figure S7b). No TOPFlash activity was detected in MM134; cotransfection with WNT1, WNT3A, or WNT4 could not induce activity. WNT1 and WNT3A activated β-catenin-dependent transcription in HT1080 cells; WNT4 did not, despite inducing phosphorylation of the DVL2/3 Wnt signaling molecules in HT1080 (Additional file 8: Figure S7c) [33]. These observations suggest that WNT4 is acting via a β-catenin-independent mechanism in ILC cells.

**Fig. 5:**
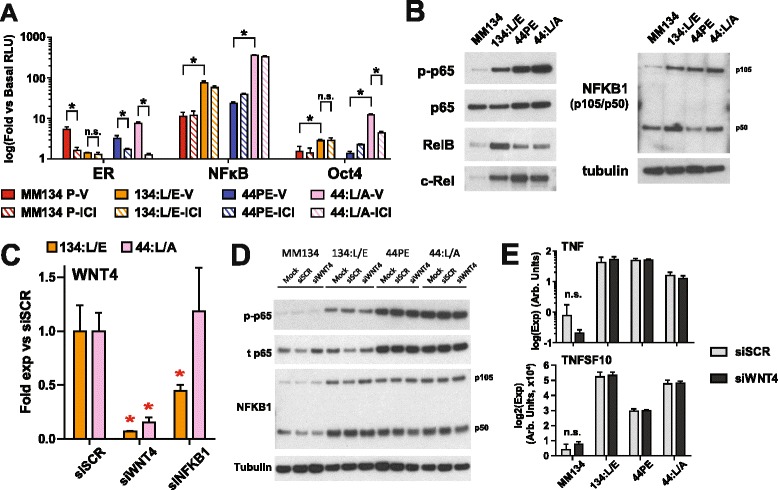
Activated nuclear factor kB (NF-kB) in invasive lobular carcinoma endocrine-resistant long-term estrogen-deprived (ILC-LTED) cells drives *WNT4* expression. **a** Transcription factor reporter arrays were performed as described in the Methods section. Data are expressed as relative luciferase units (RLU) normalized to RLU from a negative control (luciferase without a response element). *p < 0.05; n.s., not significant (*t* test). Statistical tests were not corrected for multiple comparisons in this experiment, owing to the hypothesis-generating nature of this semibiased screen. **b** Breast cancer cell lines (BCCLs) were maintained in their respective standard conditions. Immunoblotting was performed as described in the Methods section. **c** BCCLs were reverse transfected with 10 nM small interfering (siRNA), and RNA was collected 60 h posttransfection. Bars represent the mean of biological triplicate ± SD. *p < 0.05 by analysis of variance of expression vs siSCR (Dunnett’s multiple comparisons test). **d** and **e** BCCLs were reverse-transfected with 10 nM siRNA, and lysates and/or RNA were collected 48 h posttransfection. **d** Immunoblotting was performed as described in the Methods section. Images are representative of duplicate experiments. **e** Gene expression data are shown as means of biological triplicate ± SD. *p* = not significant for siSCR vs siWNT4 (*t* test). *ER* Estrogen receptor, *44PE* SUM44PE cell line, *MM134* MDA-MB-134-VI

To identify putative noncanonical pathways regulating or executing WNT4 signaling, we assessed the activity of 45 transcription factors in ILC vs ILC-LTED (parental vs 134:L/E or 44:L/A) in the presence or absence of ICI (Fig. 5a and Additional file 9: Figure S8). The ERE reporter confirmed our observations regarding endocrine response in ILC-LTED; ER activity was ablated in 134:L/E and was maintained but ICI-sensitive in 44:L/A. Among the remaining 44 reporters, two were upregulated in both ILC-LTED lines with and without ICI, nuclear factor kB (NF-kB), and Oct-4 (Fig. 5a). Oct-4 signaling was most strongly activated in 44:L/A but became ICI-sensitive in those cells. NF-kB activity was ICI-resistant and upregulated approximately seven- to tenfold in ILC-LTEDs vs parental cells. As NF-kB has previously been implicated in endocrine resistance (see Discussion section below), we further examined NF-kB signaling. Consistent with increased NF-kB activity, immunoblotting showed increased phospho-p65 and increased active NFKB1 (p50) in each ILC-LTED; notably, 134:L/E also showed increased levels of both RelB and c-Rel compared with MM134 (Fig. 5b). Increased NF-kB activity in ILC-LTED also correlated with increased sensitivity to inhibitor of NF-kB BMS vs parental cells (Additional file 10: Figure S9a). These observations demonstrate that NF-kB signaling is a critical pathway in these ILC-LTED models.

We next investigated whether NF-kB signaling is an effector or regulator of WNT4 signaling. In support of the latter, we identified two putative NF-kB/Rel binding sites [34, 35] at the WNT4 ERBS (Additional file 10: Figure S9b). Taken together with our *WNT4* expression and regulation data, we hypothesized that NF-kB signaling might be an upstream regulator responsible for the ER-independent *WNT4* upregulation in 134:LTED. To test this hypothesis, we knocked down NF-kB pathway components and assessed *WNT4* expression. In 134:L/E, knockdown of *TNF*, *TNFSF10* (*TRAIL*), *NFKB2*, and *BCL3* had no effect on *WNT4* expression (Additional file 10: Figure S9c, left); knockdown of *REL*, *RELA*, and *RELB* modestly suppressed *WNT4* expression without reaching statistical significance (Additional file 10: Figure S9c, right), potentially due to compensation among individual Rel proteins. However, targeting *NFKB1* (siNFKB1) produced approximately 50 % suppression of *WNT4* expression in 134:L/E, and this was not observed in 44:L/A (Fig. 5c). The converse of this observation was not true; siWNT4 did not affect phosphorylation of p65 or activation of p100 to p52 (Fig. 5d), nor did it affect expression of NF-kB target genes (Fig. 5e). These data suggest that though activated NF-kB was observed in both 134:LTED and 44:LTED, NF-kB signaling specifically regulated *WNT4* expression when *WNT4* was uncoupled from the ER (134:LTED), acting upstream to drive *WNT4* expression.

**Fig. 6:**
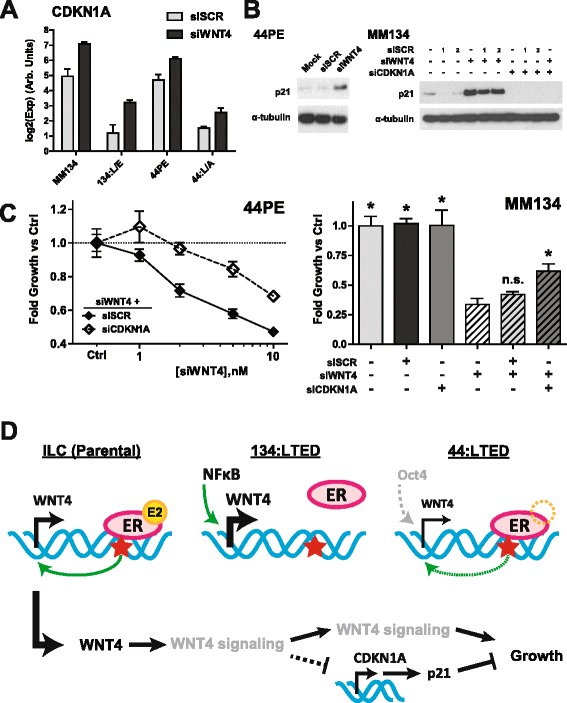
siWNT4-mediated growth suppression is mediated by increased *CDKN1A*/p21. **a** Breast cancer cell lines (BCCLs) were reverse-transfected with 10 nM small interfering RNA (siRNA), and RNA was collected 48 h posttransfection. Gene expression data are shown as the mean of biological triplicate ± SD. *p* < 0.05 for each SCR vs WNT4 (*t* test). **b** *Left*, SUM44PE (44PE) cells were reverse-transfected with 10 nM siRNA, and lysates were collected 48 h posttransfection. The samples presented were also used in Fig. 5d, and the loading control is replicated for clarity. Data are representative of duplicate experiments. *Right*, MDA-MB-134-VI (MM134) cells were reverse-transfected with 10 nM siRNA (20 nM total for combinations), and lysates were collected 48 h posttransfection. *1*, GE Dharmacon siGENOME control pool 2; *2*, Sigma-Aldrich MISSION control pool 1. Reduction in p21 protein levels with scrambled control siRNA was a class effect across all commercial scrambled siRNA pools tested, observed only in MM134 cells. **c** *Left*, 44PE cells were reverse-transfected with increasing concentrations of siWNT4 in the presence of 10 nM siSCR or siCDKN1A. Proliferation was assessed 7 days posttransfection. Data are shown as fold change vs siSCR or siCDKN1A control (no siWNT4). *p* < 0.01 by analysis of variance (ANOVA) for interaction (siSCR vs siCDKN1A on siWNT4 effect). *Right*, MM134 cells were reverse-transfected with 10 nM siRNA as indicated. Proliferation was assessed 7 days posttransfection. Data are shown as fold change vs mock transfection. *p < 0.05 by ANOVA (Dunnett’s multiple comparisons test) vs siWNT4 alone. *n.s.* Not significant. **d** Schematic of *WNT4* regulation and signaling in invasive lobular carcinoma (ILC) and ILC endocrine-resistant long-term estrogen-deprived (ILC-LTED) cells. *Red stars*, WNT4 estrogen receptor binding site. *Left*, in parental ILC cells, 17β-estradiol (E2) activates the estrogen receptor (ER), which binds at *WNT4* and drives *WNT4* expression. *Center*, in 134:LTED, the ER no longer binds at *WNT4*, and activated nuclear factor kB (NF-kB) drives increased *WNT4* expression. *Right*, In 44:LTED, the ER binds at *WNT4* despite the absence of exogenous ligands and, potentially in coordination with Oct-4, maintains weaker *WNT4* expression. *Bottom*, WNT4 initiates a Wnt signaling pathway that is likely β-catenin-independent. This leads to suppression of *CDKN1A* expression and a decrease in p21 protein, which relieves p21-mediated growth inhibition and permits cell growth.

In examining putative NF-kB target genes, we found that *CDKN1A* (p21^WAF1/CIP1^) was strongly suppressed in ILC-LTED vs parental cells, and that siWNT4 induced *CDKN1A* (Fig. 6a). Increased *CDKN1A* correlated with increased p21 protein (Fig. 6b). Regulation of *CDKN1A* was not observed in MM134 cells following treatment with β-catenin inhibitors (Additional file 10: Figure S9d), suggesting that WNT4 regulation of *CDKN1A* is not β-catenin-dependent. The fold increase in *CDKN1A* upon siWNT4 was highest in cells that were growth-inhibited by siWNT4, specifically ILC vs IDC (Additional file 10: Figure S9e). This suggests that WNT4 suppresses *CDKN1A* and that the increase in *CDKN1A*/p21 upon WNT4 knockdown leads to the inhibition of proliferation. To test this hypothesis, we combined siWNT4 with siCDKN1A to reverse the effects of siWNT4. As shown in Fig. 6c, concurrent knockdown of *CDKN1A* and *WNT4* partially reversed siWNT4-mediated growth inhibition in both 44PE and MM134. On the basis of these observations, suppression of *CDKN1A* may be a critical component of WNT4 signaling in ILC (Fig. 6d). This pathway may be activated by NF-kB signaling during the development of endocrine resistance to maintain *WNT4* expression, *CDKN1A* suppression, and cell proliferation.

### WNT4 expression is increased in luminal breast tumors

**Fig. 7:**
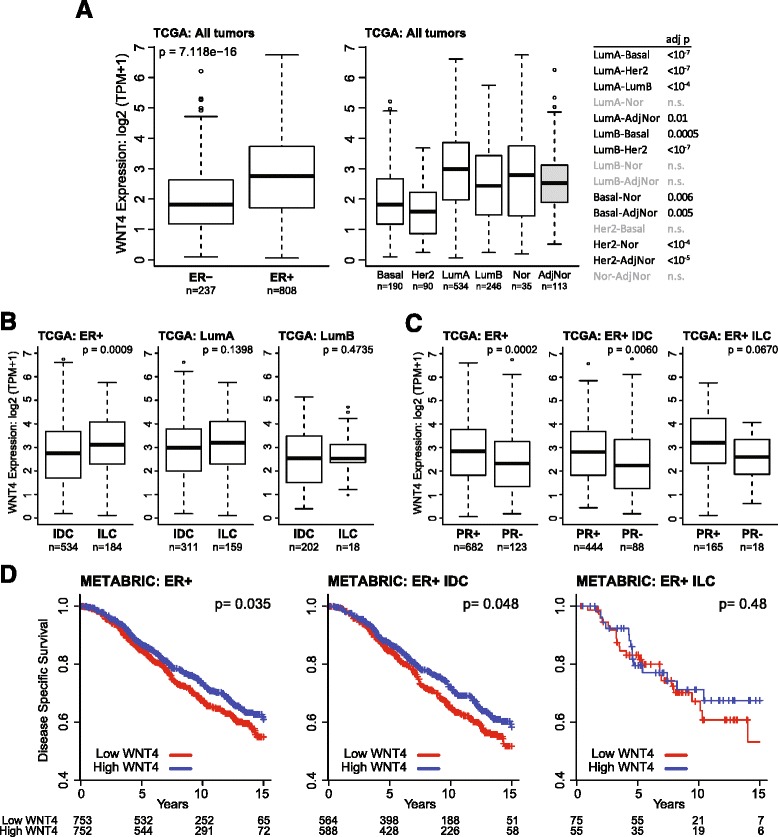
*WNT4* expression is increased in hormone receptor-positive and luminal breast tumors. Source data are described in the Methods section. **a** *Left*, *p* value by Mann-Whitney *U* test for estrogen receptor (ER)-negative vs ER-positive breast tumors. *Right*, Categories represent PAM50 molecular subtypes. *AdjNor* Adjacent normal breast tissue. *p* Values by Tukey’s test for pairwise comparisons; *n.s.* Not significant (*p* > 0.05). **b** and (**c**) *p* Values by Mann-Whitney *U* tests for invasive ductal carcinoma (IDC) vs invasive lobular carcinoma (ILC) in each subset of breast tumors. **d** *WNT4* expression groups were determined on the basis of median expression for all ER-positive breast tumors. *p* Value represents uncorrected log-rank test for low vs high *WNT4* expression. *METABRIC* Molecular Taxonomy of Breast Cancer International Consortium, *TCGA* The Cancer Genome Atlas

We used public datasets from The Cancer Genome Atlas [22] and the METABRIC study [24] to assess *WNT4* expression and associations with clinical and molecular features in breast tumors. *WNT4* expression is increased in ER-positive vs ER-negative tumors overall, and is highest in the luminal A subtype (Fig. 7a). Interestingly, *WNT4* expression was also increased in normal-like tumors and adjacent normal breast tissue vs the basal and HER2 subtypes, consistent with the role of WNT4 in normal breast physiology. Across ER-positive breast tumors, *WNT4* expression was higher in ILC than in IDC, but this may be due to the increased proportion of luminal A tumors among ILCs (Fig. 7b) [3]. *WNT4* expression was also increased in PR-positive tumors vs PR-negative tumors among all ER-positive tumors and ER-positive IDCs (Fig. 7c). A similar trend was observed in ER-positive ILCs despite the limited number of PR-negative ILCs. Consistent with increased *WNT4* expression in luminal A/PR-positive tumors, high *WNT4* expression is associated with improved disease-specific survival among all ER-positive tumors and ER-positive IDCs (Fig. 7d).

## Discussion

ILCs typically present with clinical biomarkers consistent with endocrine responsiveness, and nearly all patients with ILC are treated with adjuvant endocrine therapy [1, 36]. However, recent retrospective clinical trial data suggest that at least a subset of patients with ILC may have poor outcomes with endocrine therapy [6, 7], suggesting that, compared with IDC, ER biology and signaling may be unique in ILC cells. Consistent with this, our report on endocrine response in ILC model systems demonstrated that ER signals via unique transcriptional targets in ILC cells [8], which we hypothesized would mediate endocrine response in ILC. We identified the Wnt ligand WNT4 as a putative novel effector of ER signaling specifically in ILC cells, and we demonstrate in the present study that WNT4 is a driver of endocrine response and resistance in ILC (Fig. 6d).

In the murine mammary gland, Wnt4 is critical in pregnancy-induced ductal elongation and branching [10], as well as in maintenance of the progenitor cell niche [11–13]. In these contexts, *WNT4* serves as an effector of PR signaling and is directly regulated by P4/PR. We observed PR regulation of *WNT4* in the PR-positive BCCL T47D (Fig. 2a), but elucidating the mechanism that places *WNT4* under ER control in ILC is an important future direction for research. Interestingly, the EREs at the WNT4 ERBS are canonical half-EREs but deviate from the consensus full-ERE sequence (Additional file 1: Figure S1); thus, a specific transcription factor context may be required to access and/or use this site in ILC. Interestingly, potential ILC-specific modifiers of ER function were reported in recent large-scale genomic studies in which researchers identified differential expression and mutation of *FOXA1* vs *GATA3* [3] and amplification of *ESR1* [5]. However, we have not detected these aberrations in MM134 or 44PE (T. Du, K. Levine, M. J. Sikora, et al., unpublished manuscript; also see [22]), and thus *WNT4* regulation is likely mediated by other factors. Putative factors from murine tissues where *Wnt4* is hormone-regulated include Foxo1 [37], Foxc2 [38], Wt1 [39, 40], Mta3 [41], MED1 [42], and Egr1 [43]. Another putative cofactor may be YAP and/or TAZ, which cross-talk with both canonical and noncanonical Wnt signaling [44, 45]; nuclear (active) YAP is elevated in ILC tumors [46]. Finally, the activation of Oct-4 observed in ILC-LTED (Fig. 5a) paralleled *WNT4* expression in ILC-LTED cells (i.e., ICI sensitivity in 44:LTED), and thus Oct-4 may play a role in maintaining ER regulation of WNT4 in 44:LTED. Oct-4 may also connect differentiation or progenitor state to *WNT4* regulation. Perhaps consistent with this, *Wnt4* in the pubertal murine mammary gland is in fact modestly E2-induced, but it is strictly P4-induced in the adult gland [14]. Though these observations are intriguing, the Oct-4 response element used is likely promiscuous across Oct/POU family members, and thus Oct signaling in ILC-LTED may be tied to a number of related transcription factors, though Oct-4 itself has recently been implicated in endocrine resistance [47].

Noncanonical Wnt4 signaling pathways have been examined in murine tissues but are greatly context-dependent. β-Catenin-independent WNT4 signaling has not been extensively characterized in the breast or in breast cancer. In ILC cells, we identified *CDKN1A*/p21 as a novel WNT4 signaling target and demonstrated that *CDKN1A*/p21 regulation is a critical component of WNT4-mediated growth (Fig. 6). p21 has previously been shown as a direct transcriptional repressor of *Wnt4* [48], but we observed the converse, that WNT4 is an upstream regulator of *CDKN1A*. Though our transcription factor screen did not identify a putative effector of WNT4 to suppress *CDKN1A*, a number of factors and/or pathways have been reported downstream of Wnt4 in murine tissues and thus may be functioning in ILC, including p38/Jnk [49], SF-1(NR5A1) [50, 51], EAF1 and EAF2 [52, 53], Runx-1 [54], and Fst [55]. Yu et al. also demonstrated that noncanonical Wnt4 signaling could block ovariectomy-induced osteoporosis via inhibition of receptor activator of nuclear factor kB ligand-induced NF-kB signaling [56] (though we did not observe changes in NF-kB signaling upon siWNT4 in ILC-LTED; Fig. 5d and e). Additionally, Wnt4 regulates steroidogenesis in ovarian and adrenal models [57–59], which may have significant implications should this also be true in ILC. However, WNT4 signaling is clearly multifaceted, as siCDKN1A only partially rescued the growth suppression by siWNT4 (Fig. 6c). Identification of additional WNT4 target genes in ILC, as well as the WNT4 receptor and downstream signaling components, is a critical future direction for research.

Understanding the mechanism by which WNT4 activates its signaling cascade (i.e., in an autocrine vs paracrine mechanism) is an important future direction for research. A key observation from *Wnt4*-transgenic mice mammary gland studies was that though *Wnt4* knockout ablated ductal elongation and branching [10], the converse was not true; *Wnt4* overexpression did not induce hyperplasia or tumorigenesis [29]. However, researchers in an earlier study did observe that *Wnt4* overexpression induced mammary hyperplasia [60], and, taken together, these studies highlight the potential importance of the specific cell population expressing Wnt4 (discussed in [29]). Interestingly, studies that have demonstrated the role of Wnt4 in maintenance of the progenitor cell niche [11–13] clearly showed a paracrine role for Wnt4, wherein PR-positive, Wnt4-expressing cells secrete Wnt4 to activate signaling in neighboring hormone receptor-negative cells. Additionally, regulation of *Wnt4* may be modified during pregnancy, wherein E2 and P4 may cooperate to induce paracrine Wnt4 signaling [61]. It is unclear whether similar mechanisms may be maintained in ILC cells, whether ILC-derived WNT4 can signal in a paracrine mechanism with the tumor microenvironment, or whether WNT4 operates in a cell-autonomous vs nonautonomous manner to drive proliferation of tumor cells.

Studies examining endocrine resistance mechanisms specifically in ILC are in their infancy [1, 62]. Beyond our LTED models, only one other ILC acquired endocrine resistance model has been characterized: SUM44/LCCTam, which is a tamoxifen-resistant variant of 44PE [63]. Intriguingly, *WNT4* is among the top 50 differentially expressed genes between LCCTam and 44PE (upregulated more than twofold in LCCTam vs 44PE [GEO:GSE12708]), suggesting that WNT4 may be a common mechanism of acquired endocrine resistance in ILC cells. We further characterized WNT4-mediated endocrine resistance in ILC-LTED and identified that WNT4 signaling is maintained via activation of NF-kB signaling in 134:LTED. Activation of NF-kB is a driver of antiestrogen resistance in MCF-7 models of acquired [64], Akt-driven [65], and HER2-driven [66] resistance. In these contexts, ER is a repressor of NF-kB activity [67], and loss of canonical ER activity (and parallel loss of chicken ovalbumin upstream promoter transcription factor II [68]) leads to reactivation of NF-kB. This inverse correlation between ER and NF-kB activity has also been observed in patient tumor samples (reviewed by Sas et al. [69]). However, it does not appear that NF-kB activity is downstream of ER in ILC cells, as ICI treatment had minimal or no effect on reporter output in ILC parental or LTED cells (Fig. 5a). Thus, though NF-kB may be a shared endocrine resistance mechanism in breast cancers, the mechanism of activation and potentially its signaling may differ in IDC vs ILC. The presence of NF-kB/Rel binding sites at the WNT4 ERBS (Additional file 10: Figure S9b) suggests that *WNT4* may be a direct target of NF-kB signaling in ILC, and future studies will elucidate the context required for NF-kB to regulate *WNT4*.

The increased expression of *WNT4* in ER-positive breast tumors is consistent with the role of WNT4 in mediating hormone response in the normal mammary gland, and with our observations regarding the role of WNT4 in endocrine response in ILC cells. Though we observed ER regulation of WNT4 specifically in ILC cells, the association of *WNT4* expression with PR status across ER-positive tumors suggests that PR may regulate *WNT4* in IDC, as we observed in T47D cells, or that *WNT4* may be a marker of functional ER signaling. Importantly, the expression data currently available represent static, pretreatment measurement of *WNT4* expression in breast tumors; regulation of *WNT4* expression following endocrine therapy may be a superior biomarker for ILC biology. Though gene expression data from ILC tumors following neoadjuvant letrozole therapy have been reported [70], the expression of many ILC-specific ER target genes [8], including *WNT4*, were excluded from the analyses due to issues related to the use of multiple expression platforms. Future analyses of *WNT4* regulation may be possible on the basis of ongoing studies such as POETIC [71] or our neoadjuvant trial for patients with ILC (ClinicalTrials.gov identifier NCT02206984 [72]).

## Conclusions

Recent clinical and laboratory studies suggest that despite biomarkers consistent with favorable response to endocrine therapy, ILC cells may use unique ER signaling pathways to mediate endocrine response and resistance. We have identified the Wnt ligand WNT4 as a novel, critical effector of ER signaling in ILC cells, which co-opt a PR-driven developmental pathway by placing it under direct ER control. ER regulation of *WNT4* correlates with use of an ERBS at the *WNT4* locus specifically in ILC cells. Novel endocrine-resistant ILC-LTED models either maintain ER function and *WNT4* regulation or uncouple *WNT4* from ER and upregulate *WNT4* expression. Activated NF-kB signaling can drive this upregulation of *WNT4* in ILC-LTED cells. In both ILC and ILC-LTED cells, WNT4 suppresses *CDKN1A*/p21, which is critical for maintenance of ILC cell proliferation. Knockdown of *CDKN1A* can partially reverse the effects of siWNT4. Taken together, these observations demonstrate that WNT4 drives a critical signaling pathway in mediating endocrine response and resistance in ILC. Future studies will examine the mechanisms leading to ER control of *WNT4* expression, elucidate the components of the WNT4 signaling pathway, and evaluate the role of this pathway in tumor models systems including patient-derived xenografts and primary tumor tissue explants.

## Additional files

Additional file 1: Figure S1.
*WNT4* is a putative direct ER target gene in ILC cells. A schematic of WNT4 ERBS is shown. ER binding at *WNT4* was observed in MM134, but not in IDC cells [73]. Sequence of predicted EREs (*red star*) is shown vs consensus ERE. *Red letters* represent a match with consensus. MM134 ChIP-seq data are derived from the study by Sikora et al. [8]. *MACS* Model-based analysis of ChIP-Seq. (PDF 149 kb) Additional file 2: Sequence and product information for oligos used in this study. (XLSX 11 kb) Additional file 3: Figure S2. Extent of *WNT4* knockdown correlates with effect of cell proliferation. MM134 cells were reverse-transfected with increasing concentrations of MISSION siWNT4 pool (Sigma-Aldrich) or siGENOME siWNT4 pool (GE Dharmacon, Lafayette, CO, USA) or were treated with 1 μM ICI. Ctrl, Nontransfected; Rgt, Reagent-only (mock)-transfected. RNA was harvested 48 h posttransfection, and proliferation was assessed 6 days posttransfection. In each case, data are normalized to Ctrl (100 %) and ICI-treated (0 %). (PDF 89 kb) Additional file 4: Figure S3. SUM44/F has improved endocrine response vs 44PE. SUM44/F cells were derived from 44PE as described in the Methods section. Each line was hormone-deprived prior to treatment with vehicle (0.1 % EtOH) or increasing concentrations of E2. Proliferation was assessed 6 days posttreatment. Growth is shown as fold change vs vehicle control. (PDF 87 kb) Additional file 5: Figure S4. ER regulation of *WNT4* and potential interaction with PR at binding sites near the *WNT4* gene locus. **a** MM330 cells were hormone-deprived as described in Materials and Methods and treated with 1 nM E2 ± 1 μM ICI for 24 h. Bars represent biological triplicate ± SD. **p* < 0.05 by one-way analysis of variance vs respective control with Dunnett’s multiple comparisons test. **b** Data derived from the study by Mohammed et al. [27] [GEO:GSE68355]. Model-based analysis of ChIP-Seq (MACS) peaks were visualized using the Integrated Genomics Viewer browser (Broad Institute, Cambridge, MA, USA), and aligned the RefSeq gene set in hg19. *Dashed green box* is an enlargement showing binding sites proximal to the WNT4 ERBS in MM134 and the *WNT4* transcriptional start site. *Dashed blue box* shows an upstream region (−30 kb) of P4-induced ER/PR binding in MCF-7. **c** Samples were treated and ChIP-qPCR performed as described in Fig. 2 legend. *IgG* Immunoglobulin G. (PDF 175 kb) Additional file 6: Figure S5. Establishment of long-term estrogen-deprived variants of ILC cell lines. **a** Schematic representation of development of LTED lines together with representative phase-contrast images (original magnification × 100) of resulting lines. **b** Once established (i.e., able to be passaged routinely in T75 flasks), lysates were collected from LTED lines, parental ILC cells, and MCF-7 cells. Immunoblotting was performed as described in the Methods section. **c** Endocrine response was assessed in ILC-LTED vs hormone-deprived parental cells. BCCL cells were treated as indicated, and proliferation was assessed 6 days after treatment. Proliferation is shown as baseline subtracted vs vehicle (0.01 % EtOH) treatment. **p* < 0.05 treatment vs vehicle control (*t* test). ^+^Immediately after establishment, 134:L/B maintained modest endocrine responsiveness, but within several passages this was lost. Repeat experiments revealed no difference between treatment and vehicle. *IMEM* Improved minimal essential medium. (PDF 198 kb) Additional file 7: Figure S6. ER and FGFR signaling in ILC-LTED cells. **a** BCCL cells were treated and processed as described in the Fig. 3 legend. Bars represent mean of biological triplicate ± SD. **p* < 0.05 for vehicle control vs ICI (*t* test). “E2 status” denotes the hormone status of the experimental culture medium. ^+^FBS-containing medium; ^−^CSS-containing medium. **b** ILC-LTED cells were reverse-transfected with 10 nM siSCR or siWNT4 and allowed to attached overnight; cells were then treated with increasing concentrations of FGFR inhibitor PD173074. Growth was assessed 4 days and 5 days posttreatment for 134:L/E and 44:L/A, respectively. (PDF 113 kb) Additional file 8: Figure S7. β-Catenin dysfunction leads to lack of canonical Wnt signaling in ILC cells. **a** Reverse-phase protein array data derived from TCGA were extracted from the cBioPortal for Cancer Genomics. **b** HT1080 (Wnt-responsive fibrosarcoma cells [33]) or MM134 cells were cotransfected with TOPFlash and Renilla luciferase reporter plasmids, along with the indicated WNT plasmid or enhanced green fluorescent protein (EGFP). EGFP plasmid was included at 5 % of plasmid mass in all transfections to allow for visual confirmation of transfection. Twenty-four hours posttransfection, lysates were harvested for luciferase detection. RLU represent fold change vs EGFP cotransfection control. Bars represent mean of biological triplicate ± SD. **c** Parental HT1080 cells and HT1080-expressing WNT4 were assayed for DVL activation by immunoblotting. The size shifts of DVL2/3 are consistent with protein phosphorylation and Wnt signaling activation [33]. (PDF 128 kb) Additional file 9: Figure S8. Cignal 45-Reporter Array identifies activated signaling pathways in ILC-LTED cells. Transcription factor reporter arrays were performed as described in the Methods section. Luciferase output is shown as raw RLU. *Dashed lines* at −6 are for visual reference only. ^<^ *p* < 0.05 for vehicle vs ICI (*t* test); ^−^not significant. ^x^ *p* < 0.05 for LTED vs parental (for vehicle-treated) (*t* test). Note that statistical tests were not corrected for multiple comparisons in this experiment, owing to the hypothesis-generating nature of this semibiased screen. (PDF 153 kb) Additional file 10: Figure S9. NF-kB signaling in ILC-LTED drives WNT4-*CDKN1A* regulation. **a** *Left*, BCCL cells were treated with increasing concentrations of BMS-345541. Proliferation was assessed 6 days after treatment, shown as fold change vs vehicle (0.1 % dimethyl sulfoxide) control. **p* < 0.0001 for parental vs LTED proliferation at 10 μM BMS (*t* test). *Right*, BCCL cells were treated with increasing concentrations of BMS. Lysates were collected 48 h posttreatment. Differential sensitivity to growth suppression at 10 μM BMS correlates with ablation of p-p65 at this concentration. **b** Schematic of WNT4 ERBS indicating location and sequence of predicted NF-kB/Rel binding sites. Consensus RELA (p65) and NFKB1 binding sites are shown for reference. **c** 134:L/E cells were reverse-transfected with 10 nM siRNA (individual constructs; numbers reference those shown in Additional file 2), and RNA was collected 60 h posttransfection. Bars represent the mean of biological triplicate ± SD. **p* < 0.05 by ANOVA for expression vs siSCR (Dunnett’s multiple comparisons test). **d** MM134 cells were reverse-transfected with 10 nM siWNT4 or treated with 1 μM IWR or 10 μM JW for 24 h. Bars represent biological triplicate ± SD. **p* < 0.05 by ANOVA (Dunnett’s multiple comparisons test) vs control. **e** MCF-7 and HCC1428 cells were treated and processed as described in the Fig. 5e legend. ILC and ILC-LTED data are reproduced from Fig. 5e. Data are normalized to siSCR control knockdown. (PDF 218 kb)

## Abbreviations

ANOVA: Analysis of variance
ATCC: American Type Culture Collection
BMS: BMS-345541
44PE: SUM44PE
4-OHT: 4-Hydroxytamoxifen
BCCL: Breast cancer cell line
ChIP: Chromatin immunoprecipitation
CSS: Charcoal-stripped FBS
E2: 17β-Estradiol
EGFP: Enhanced green fluorescent protein
ER: Estrogen receptor
ERBS: Estrogen receptor binding site
ERE: Estrogen response element
FGFR: Fibroblast growth factor receptor
HER2: Human epidermal growth factor receptor 2
ICI: ICI 182,780, Fulvestrant
IDC: Invasive ductal carcinoma
IgG: Immunoglobulin G
ILC: Invasive lobular carcinoma
IMEM: Improved minimal essential medium
IWR: *Endo*-IWR1
JW: JW 67
LTED: Long-term estrogen deprivation
METABRIC: Molecular Taxonomy of Breast Cancer International Consortium
MM134: MDA-MB-134-VI
NF-kB: Nuclear factor kB
P4: progesterone
PCR: Polymerase chain reaction
PR: Progesterone receptor
qPCR: Quantitative polymerase chain reaction
RLU: Relative luciferase units
RU: RU486, Mifepristone
siRNA: Small interfering RNA
siSCR: Scrambled siRNA control
STS: Staurosporine
TCGA: The Cancer Genome Atlas
TSE: Tris-sucrose-ethylenediaminetetraacetic acid
