# Supplementary figures and images for: WNT4 mediates estrogen receptor signaling and endocrine resistance in invasive lobular carcinoma cell lines
Source: Breast Cancer Res. 2016 Sep 20;18:92. doi: 10.1186/s13058-016-0748-7 (PMC5028957; doi:10.1186/s13058-016-0748-7)

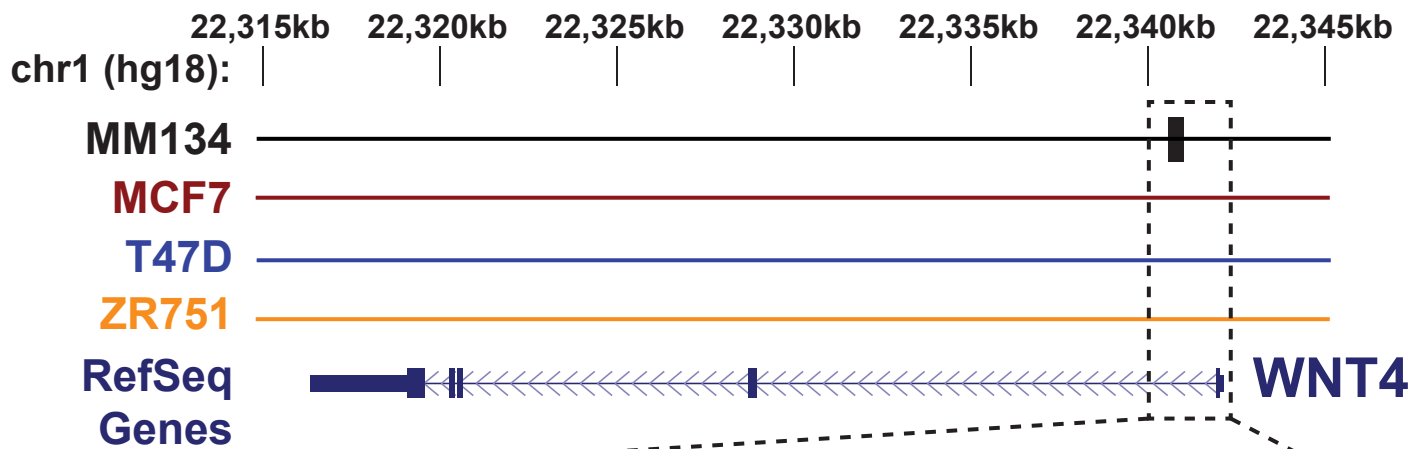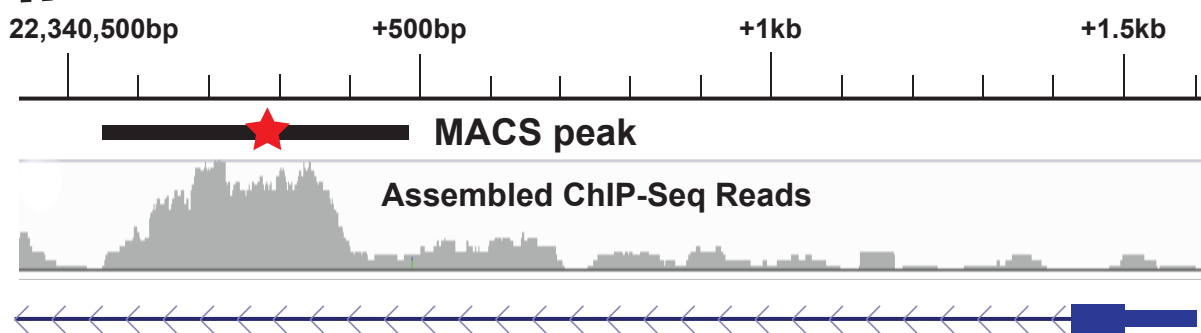

...cctG**GGT**GGctc**TGACCC**...(37bp)...T**GGCCA**caa**TGACCT**cat...

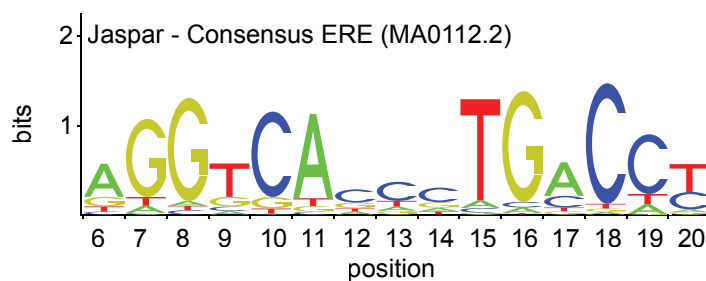

Supplement: Additional file 1: Figure S1. — WNT4 is a putative direct ER target gene in ILC cells. A schematic of WNT4 ERBS is shown. ER binding at WNT4 was observed in MM134, but not in IDC cells [73]. Sequence of predicted EREs (red star) is shown vs consensus ERE. Red letters represent a match with consensus. MM134 ChIP-seq data are derived from the study by Sikora et al. [8]. MACS Model-based analysis of ChIP-Seq. (PDF 149 kb) [file 13058_2016_748_MOESM1_ESM.pdf]

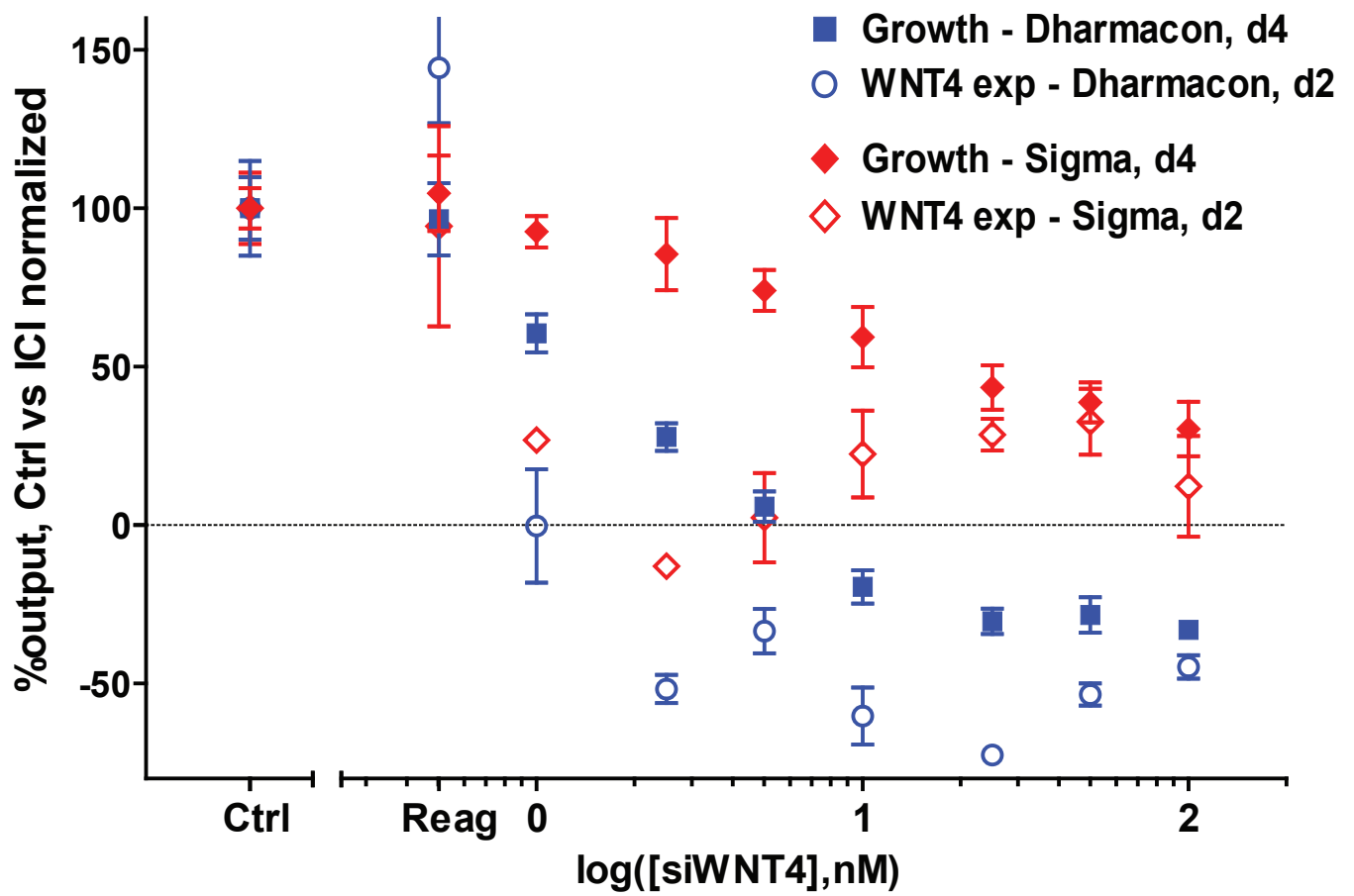

Supplement: Additional file 3: Figure S2. — Extent of WNT4 knockdown correlates with effect of cell proliferation. MM134 cells were reverse-transfected with increasing concentrations of MISSION siWNT4 pool (Sigma-Aldrich) or siGENOME siWNT4 pool (GE Dharmacon, Lafayette, CO, USA) or were treated with 1 μM ICI. Ctrl, Nontransfected; Rgt, Reagent-only (mock)-transfected. RNA was harvested 48 h posttransfection, and proliferation was assessed 6 days posttransfection. In each case, data are normalized to Ctrl (100 %) and ICI-treated (0 %). (PDF 89 kb) [file 13058_2016_748_MOESM3_ESM.pdf]

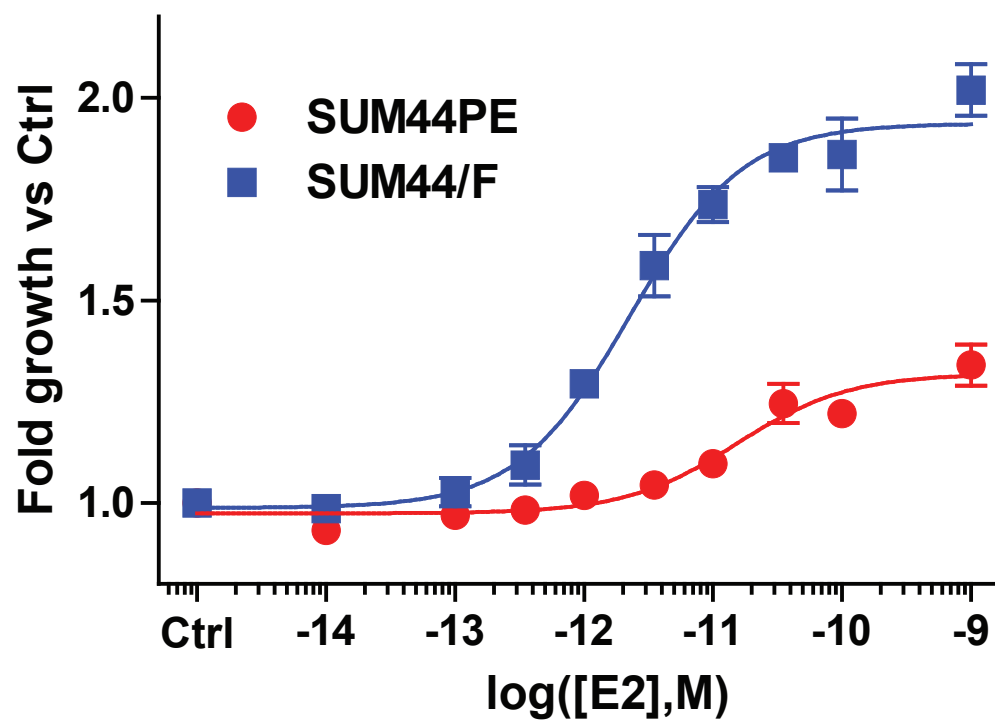

Supplement: Additional file 4: Figure S3. — SUM44/F has improved endocrine response vs 44PE. SUM44/F cells were derived from 44PE as described in the Methods section. Each line was hormone-deprived prior to treatment with vehicle (0.1 % EtOH) or increasing concentrations of E2. Proliferation was assessed 6 days posttreatment. Growth is shown as fold change vs vehicle control. (PDF 87 kb) [file 13058_2016_748_MOESM4_ESM.pdf]

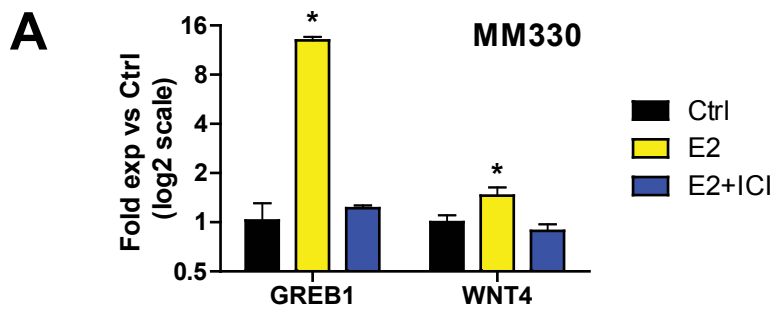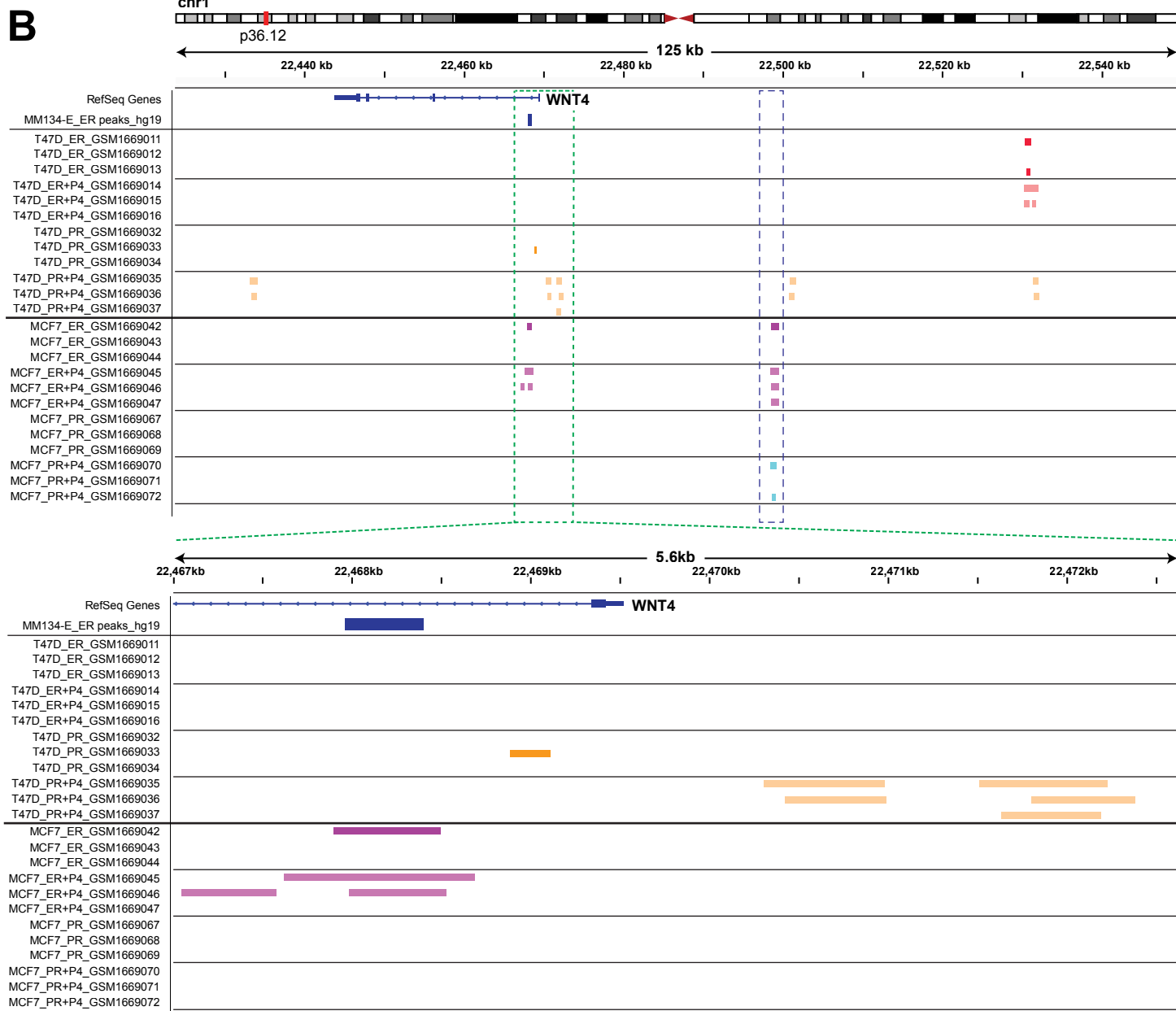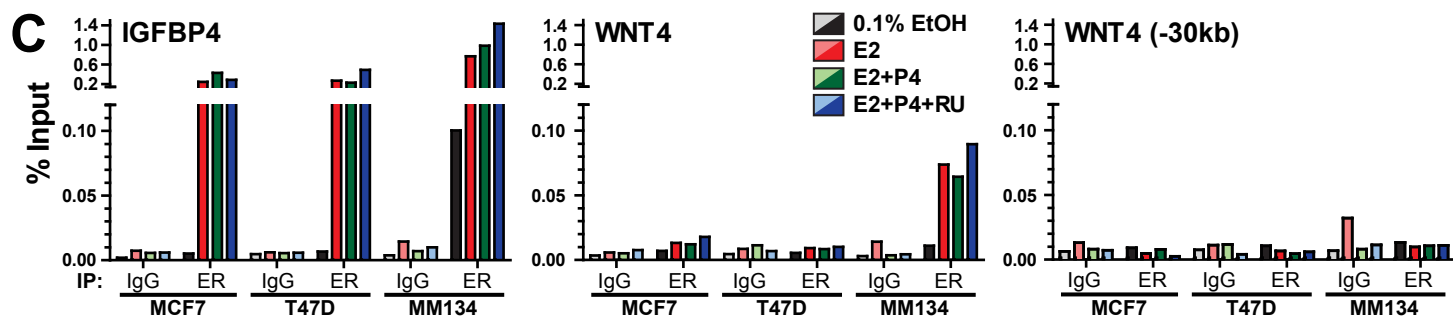

Supplement: Additional file 5: Figure S4. — ER regulation of WNT4 and potential interaction with PR at binding sites near the WNT4 gene locus. a MM330 cells were hormone-deprived as described in Materials and Methods and treated with 1 nM E2 ± 1 μM ICI for 24 h. Bars represent biological triplicate ± SD. *p < 0.05 by one-way analysis of variance vs respective control with Dunnett’s multiple comparisons test. b Data derived from the study by Mohammed et al. [27] [GEO:GSE68355]. Model-based analysis of ChIP-Seq (MACS) peaks were visualized using the Integrated Genomics Viewer browser (Broad Institute, Cambridge, MA, USA), and aligned the RefSeq gene set in hg19. Dashed green box is an enlargement showing binding sites proximal to the WNT4 ERBS in MM134 and the WNT4 transcriptional start site. Dashed blue box shows an upstream region (−30 kb) of P4-induced ER/PR binding in MCF-7. c Samples were treated and ChIP-qPCR performed as described in Fig. 2 legend. IgG Immunoglobulin G. (PDF 175 kb) [file 13058_2016_748_MOESM5_ESM.pdf]

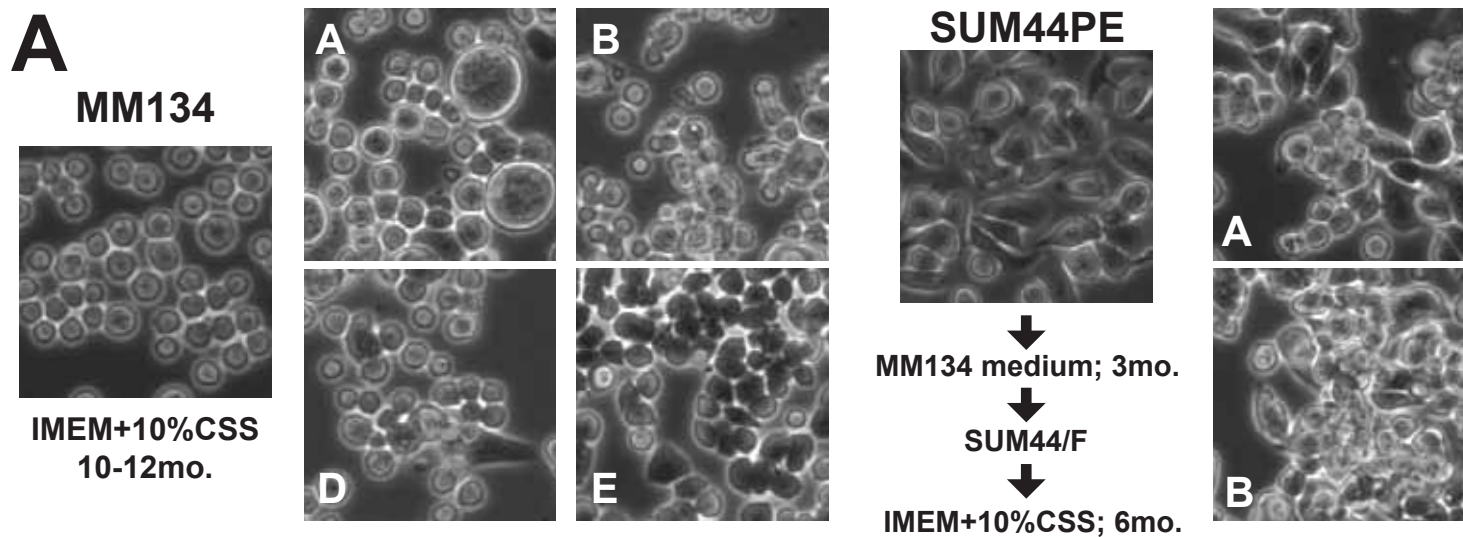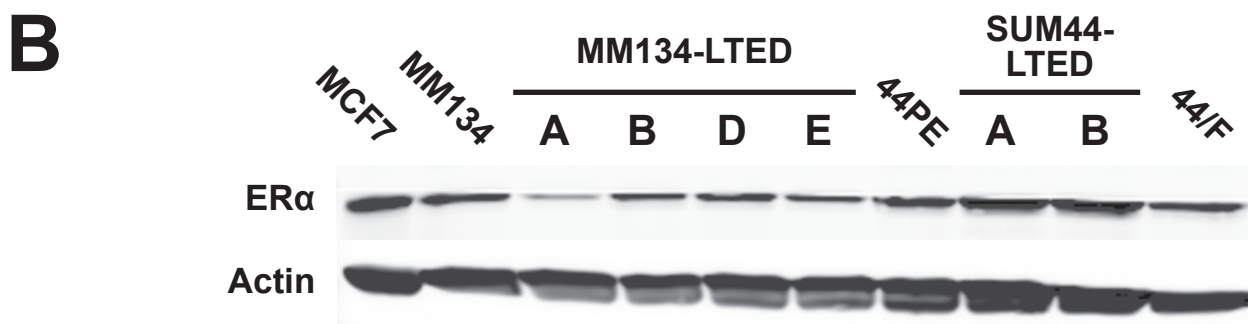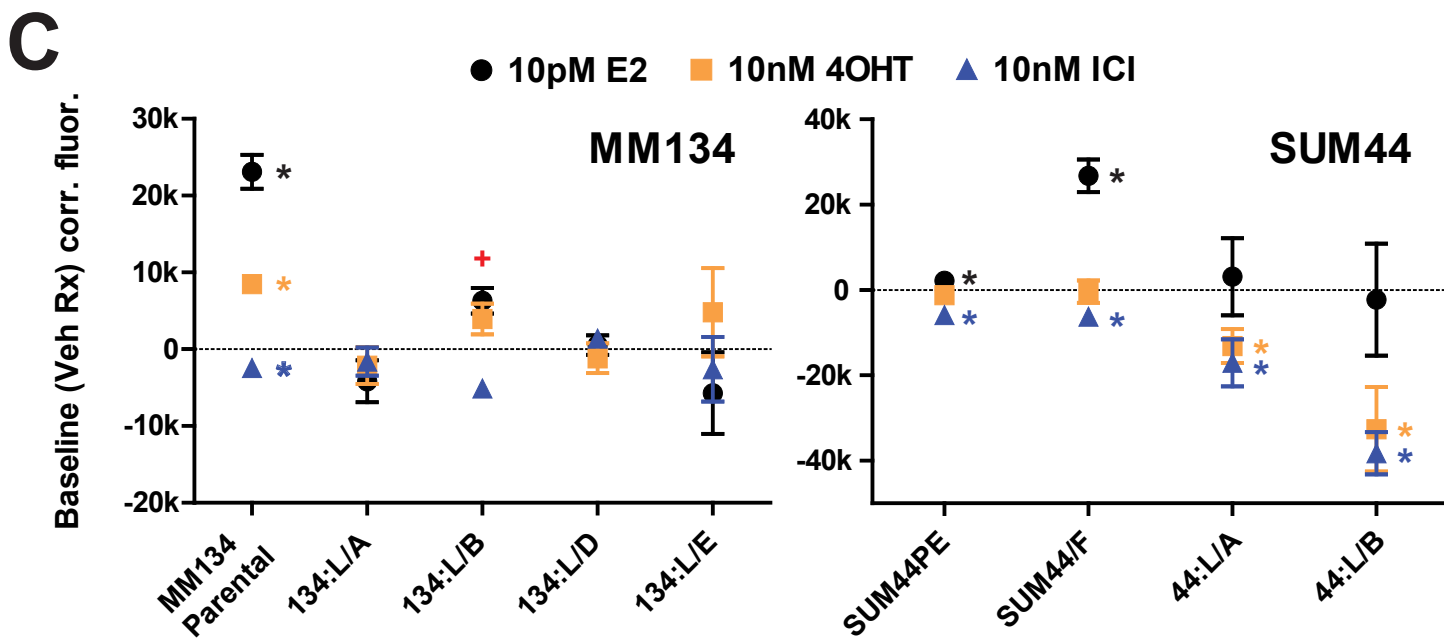

Supplement: Additional file 6: Figure S5. — Establishment of long-term estrogen-deprived variants of ILC cell lines. a Schematic representation of development of LTED lines together with representative phase-contrast images (original magnification × 100) of resulting lines. b Once established (i.e., able to be passaged routinely in T75 flasks), lysates were collected from LTED lines, parental ILC cells, and MCF-7 cells. Immunoblotting was performed as described in the Methods section. c Endocrine response was assessed in ILC-LTED vs hormone-deprived parental cells. BCCL cells were treated as indicated, and proliferation was assessed 6 days after treatment. Proliferation is shown as baseline subtracted vs vehicle (0.01 % EtOH) treatment. *p < 0.05 treatment vs vehicle control (t test). +Immediately after establishment, 134:L/B maintained modest endocrine responsiveness, but within several passages this was lost. Repeat experiments revealed no difference between treatment and vehicle. IMEM Improved minimal essential medium. (PDF 198 kb) [file 13058_2016_748_MOESM6_ESM.pdf]

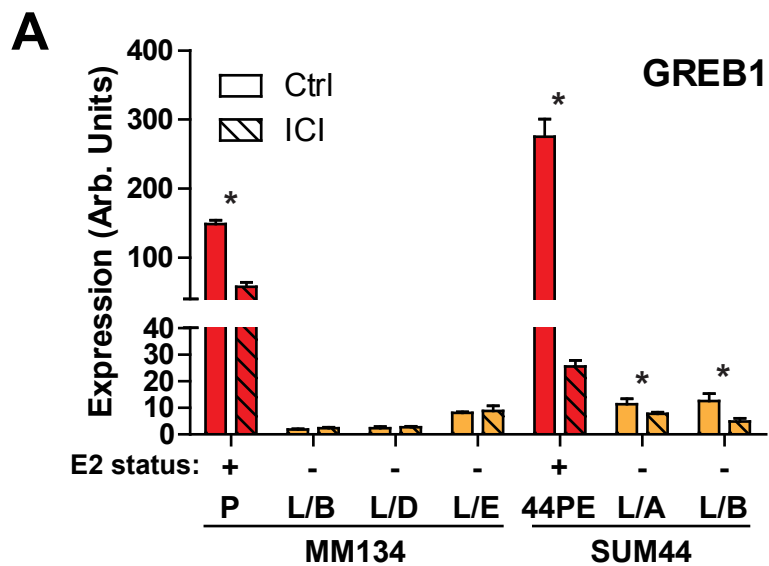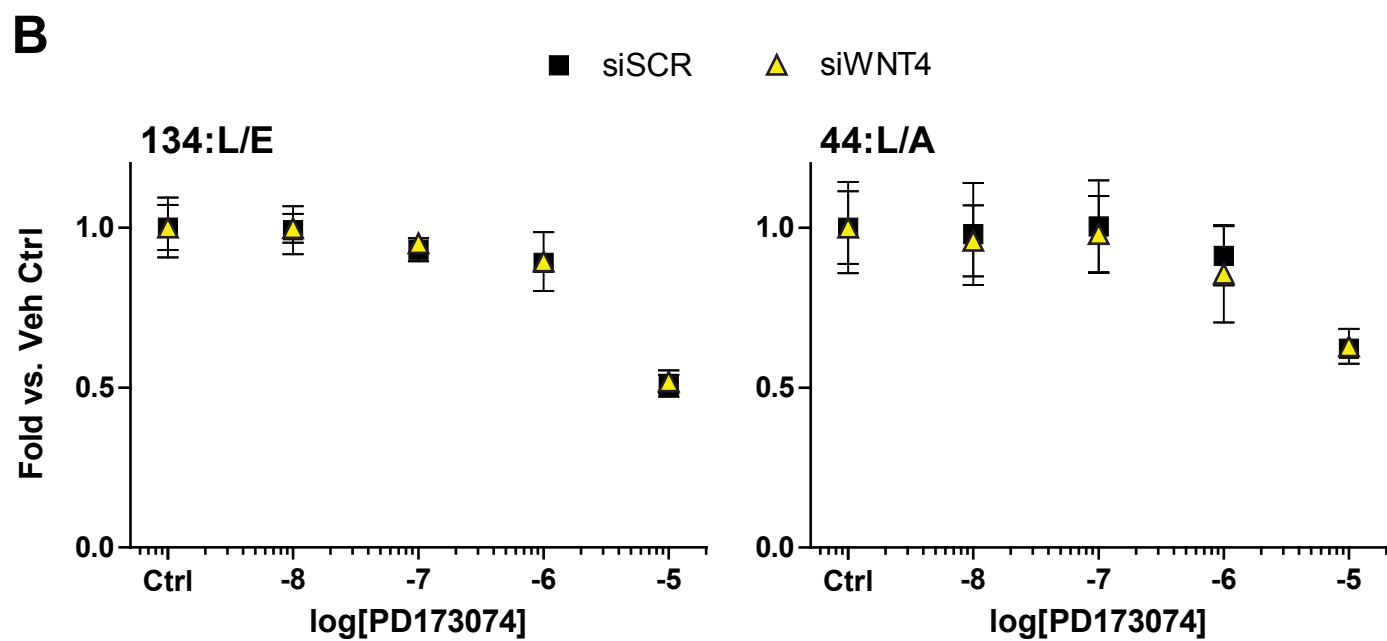

Supplement: Additional file 7: Figure S6. — ER and FGFR signaling in ILC-LTED cells. a BCCL cells were treated and processed as described in the Fig. 3 legend. Bars represent mean of biological triplicate ± SD. *p < 0.05 for vehicle control vs ICI (t test). “E2 status” denotes the hormone status of the experimental culture medium. +FBS-containing medium; −CSS-containing medium. b ILC-LTED cells were reverse-transfected with 10 nM siSCR or siWNT4 and allowed to attached overnight; cells were then treated with increasing concentrations of FGFR inhibitor PD173074. Growth was assessed 4 days and 5 days posttreatment for 134:L/E and 44:L/A, respectively. (PDF 113 kb) [file 13058_2016_748_MOESM7_ESM.pdf]

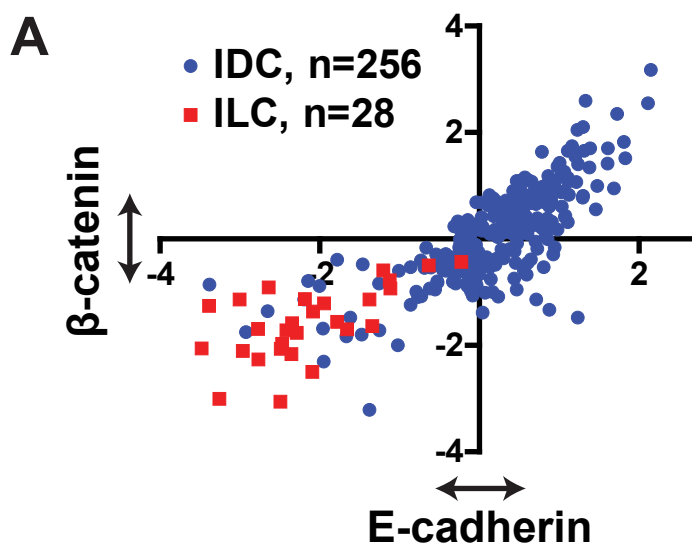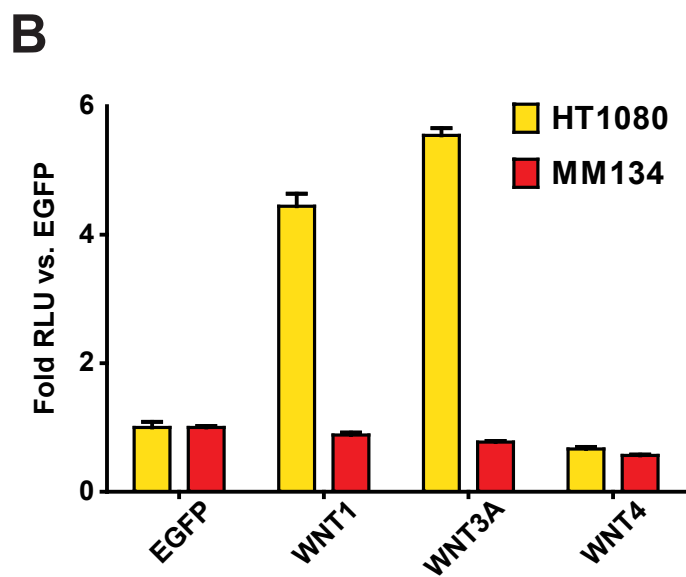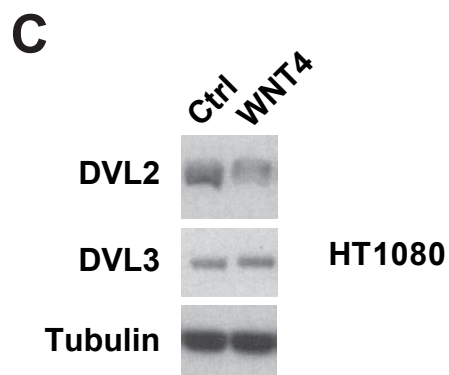

Supplement: Additional file 8: Figure S7. — β-Catenin dysfunction leads to lack of canonical Wnt signaling in ILC cells. a Reverse-phase protein array data derived from TCGA were extracted from the cBioPortal for Cancer Genomics. b HT1080 (Wnt-responsive fibrosarcoma cells [33]) or MM134 cells were cotransfected with TOPFlash and Renilla luciferase reporter plasmids, along with the indicated WNT plasmid or enhanced green fluorescent protein (EGFP). EGFP plasmid was included at 5 % of plasmid mass in all transfections to allow for visual confirmation of transfection. Twenty-four hours posttransfection, lysates were harvested for luciferase detection. RLU represent fold change vs EGFP cotransfection control. Bars represent mean of biological triplicate ± SD. c Parental HT1080 cells and HT1080-expressing WNT4 were assayed for DVL activation by immunoblotting. The size shifts of DVL2/3 are consistent with protein phosphorylation and Wnt signaling activation [33]. (PDF 128 kb) [file 13058_2016_748_MOESM8_ESM.pdf]

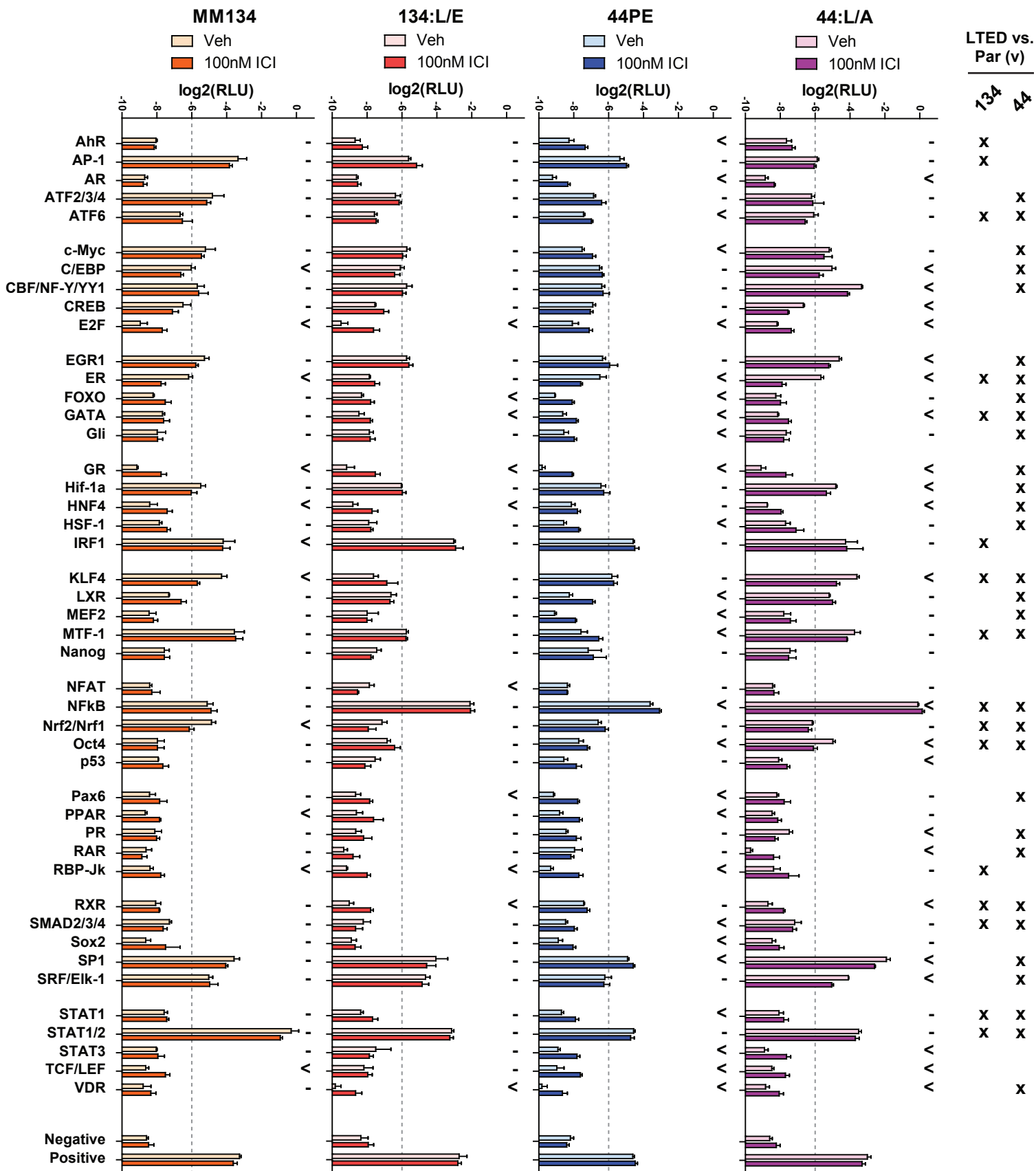

Supplement: Additional file 9: Figure S8. — Cignal 45-Reporter Array identifies activated signaling pathways in ILC-LTED cells. Transcription factor reporter arrays were performed as described in the Methods section. Luciferase output is shown as raw RLU. Dashed lines at −6 are for visual reference only. < p < 0.05 for vehicle vs ICI (t test); −not significant. x p < 0.05 for LTED vs parental (for vehicle-treated) (t test). Note that statistical tests were not corrected for multiple comparisons in this experiment, owing to the hypothesis-generating nature of this semibiased screen. (PDF 153 kb) [file 13058_2016_748_MOESM9_ESM.pdf]

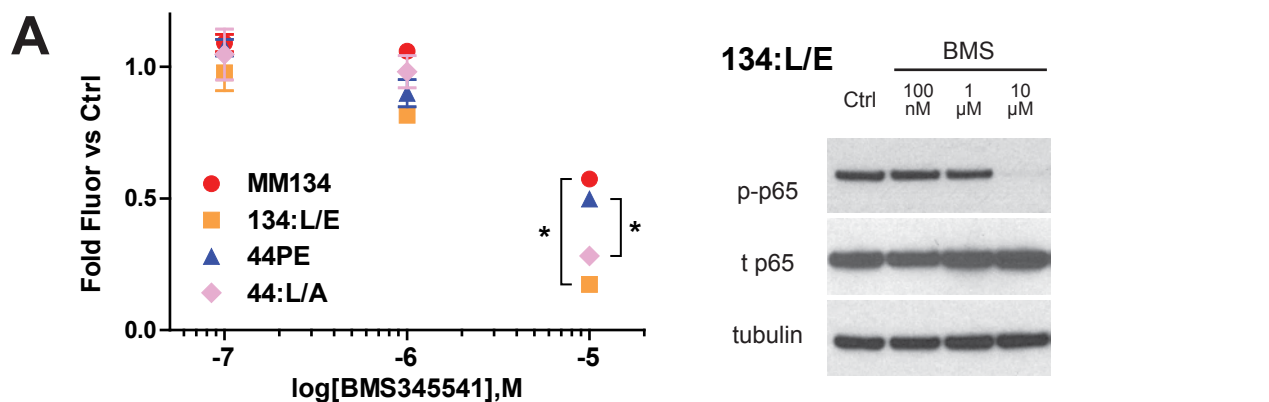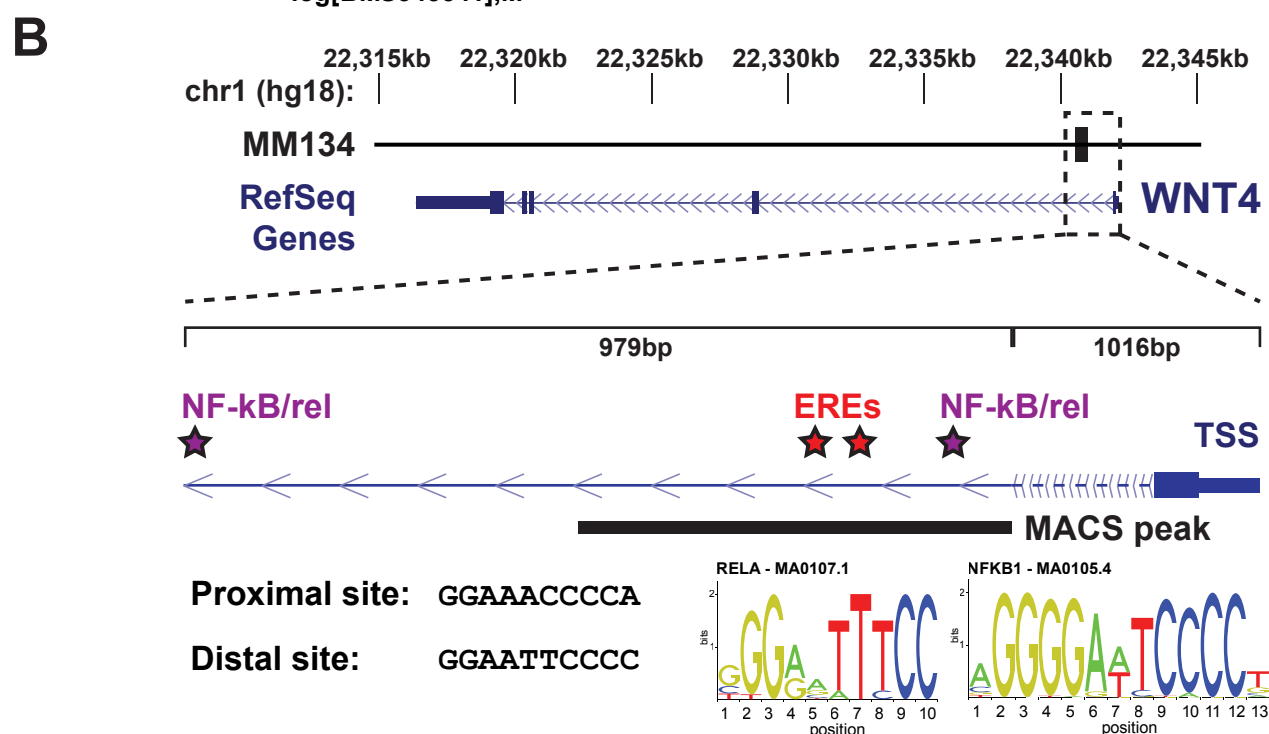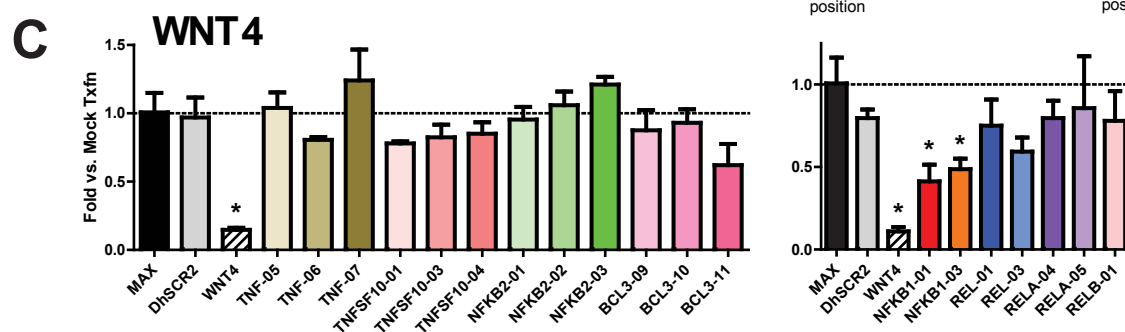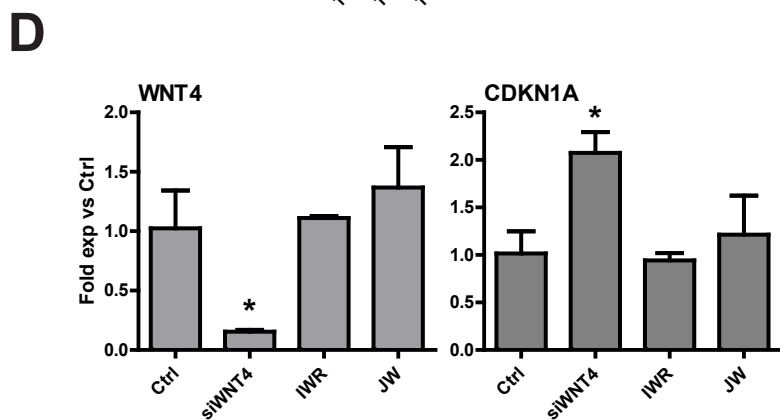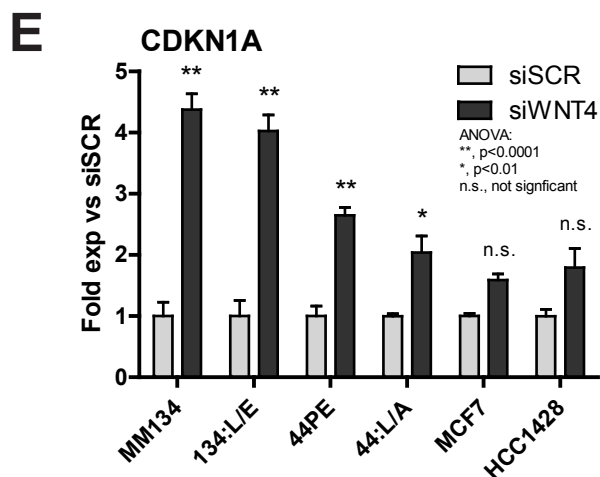

Supplement: Additional file 10: Figure S9. — NF-kB signaling in ILC-LTED drives WNT4-CDKN1A regulation. a Left, BCCL cells were treated with increasing concentrations of BMS-345541. Proliferation was assessed 6 days after treatment, shown as fold change vs vehicle (0.1 % dimethyl sulfoxide) control. *p < 0.0001 for parental vs LTED proliferation at 10 μM BMS (t test). Right, BCCL cells were treated with increasing concentrations of BMS. Lysates were collected 48 h posttreatment. Differential sensitivity to growth suppression at 10 μM BMS correlates with ablation of p-p65 at this concentration. b Schematic of WNT4 ERBS indicating location and sequence of predicted NF-kB/Rel binding sites. Consensus RELA (p65) and NFKB1 binding sites are shown for reference. c 134:L/E cells were reverse-transfected with 10 nM siRNA (individual constructs; numbers reference those shown in Additional file 2), and RNA was collected 60 h posttransfection. Bars represent the mean of biological triplicate ± SD. *p < 0.05 by ANOVA for expression vs siSCR (Dunnett’s multiple comparisons test). d MM134 cells were reverse-transfected with 10 nM siWNT4 or treated with 1 μM IWR or 10 μM JW for 24 h. Bars represent biological triplicate ± SD. *p < 0.05 by ANOVA (Dunnett’s multiple comparisons test) vs control. e MCF-7 and HCC1428 cells were treated and processed as described in the Fig. 5e legend. ILC and ILC-LTED data are reproduced from Fig. 5e. Data are normalized to siSCR control knockdown. (PDF 218 kb) [file 13058_2016_748_MOESM10_ESM.pdf]
